# Large language models approach clinician performance in ESC cardiovascular risk stratification: a vignette-based benchmark study

**DOI:** 10.1093/ehjdh/ztag073

**Published:** 2026-05-20

**Authors:** José Ferreira Santos, Regina de Brito Duarte, Inês Mota, Rita Carvalheira Santos, José Maria Moreira, Joana Campos, Nuno André Silva, Bernardo Neves, Ricardo Ladeiras-Lopes, Francisca Leite, Hélder Dores

**Affiliations:** Católica Medical School, Sintra Campus, Estrada OctávioPato, 2635-631 Rio de Mouro, Lisboa, Portugal; Cardiology Department, Setúbal, Hospital da Luz Setúbal, Luz Saúde, Estrada Nacional 10, Km 37, 2900-722 Setúbal, Portugal; Instituto Superior Técnico, Universidade de Lisboa, Lisboa, Portugal; Instituto de Engenharia de Sistemas e Computadores, Investigação e Desenvolvimento (INESC-ID), Lisboa, Portugal; Hospital da Luz Learning Health, Luz Saúde, Lisboa, Portugal; Cardiology Department, Setúbal, Hospital da Luz Setúbal, Luz Saúde, Estrada Nacional 10, Km 37, 2900-722 Setúbal, Portugal; Hospital da Luz Learning Health, Luz Saúde, Lisboa, Portugal; Instituto Superior Técnico, Universidade de Lisboa, Lisboa, Portugal; Hospital da Luz Learning Health, Luz Saúde, Lisboa, Portugal; Católica Medical School, Sintra Campus, Estrada OctávioPato, 2635-631 Rio de Mouro, Lisboa, Portugal; Hospital da Luz Learning Health, Luz Saúde, Lisboa, Portugal; Hospital da Luz Lisboa, Luz Saúde, Lisboa, Portugal; Cardiovascular Research and Development Centre-UnIC@RISE, Department of Surgery and Physiology, Faculty of Medicine of the University of Porto, Porto, Portugal; Hospital da Luz Guimarães, Luz Saúde, Guimarães, Portugal; Hospital da Luz Póvoa do Varzim, Luz Saúde, Portugal; Católica Medical School, Sintra Campus, Estrada OctávioPato, 2635-631 Rio de Mouro, Lisboa, Portugal; Hospital da Luz Learning Health, Luz Saúde, Lisboa, Portugal; Hospital da Luz Lisboa, Luz Saúde, Lisboa, Portugal; CHRC, NOVA Medical School, Lisboa, Portugal; NOVA Medical School, Lisboa, Póvoa do Varzim, Portugal; CoLAB TRIALS, Évora, Portugal

**Keywords:** Clinical decision support, Artificial intelligence, Large language models, Cardiovascular prevention, Risk stratification, SCORE2

## Abstract

**Aims:**

Guideline-based cardiovascular risk stratification requires three distinct competencies: extracting risk factor data from clinical text, computing a validated risk score, and applying guideline-defined thresholds to assign a final risk category. We evaluated contemporary large language models (LLMs) on each of these tasks within the European Society of Cardiology (ESC) SCORE2 framework and compared LLM performance against a pooled individual clinician benchmark to contextualize findings against real-world human reproducibility.

**Methods and results:**

Eleven LLMs were evaluated using 30 simulated outpatient clinical vignettes presented in both Portuguese and English. For each vignette, models extracted cardiovascular risk factors, determined SCORE2 applicability, generated 10-year risk estimates where appropriate, and assigned a final three-class ESC risk category. A committee of three cardiologists established the reference standard; eight independent clinicians provided an individual-level human benchmark. Traditional risk-factor extraction was near-perfect across all models (micro-F1 0.97–0.99). Agreement with expert-assigned final risk categories was moderate and variable (best: GPT-4o, quadratic-weighted κw 0.69, 95% CI 0.44–0.84), with 10 of 11 models more often underestimating than overestimating risk. To isolate the source of classification error, *post hoc* deterministic recalculation of SCORE2 was performed using model-extracted variables in eligible vignettes; this markedly improved agreement across all models (κw 0.85–0.90), demonstrating that extraction was largely intact and computational execution was the primary failure mode. The pooled individual clinician benchmark showed moderate agreement with the reference standard (κw 0.52, 95% CI 0.28–0.67), indicating that the best-performing LLMs matched or exceeded the average individual clinician on this guideline-based task. Performance was broadly consistent across Portuguese and English.

**Conclusion:**

Contemporary LLMs reliably extract cardiovascular risk information from clinical text, and the best-performing systems achieved agreement within the range of average individual clinicians on this structured task. Their principal limitation lies in downstream computation and rule application.

## Graphical Abstract

**Graphical Abstract:**
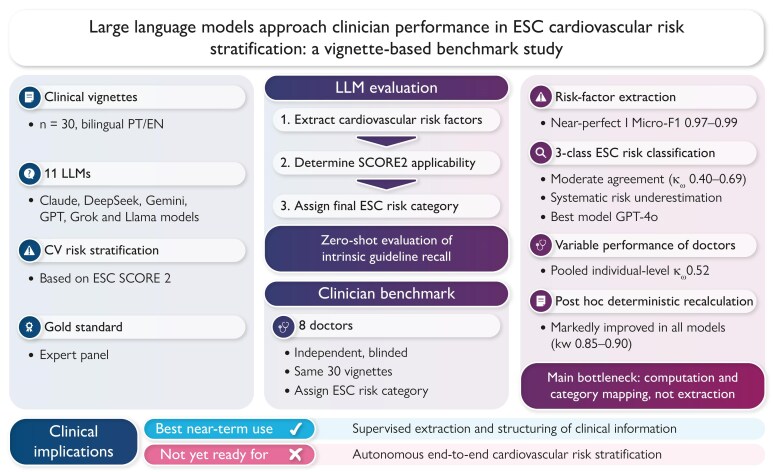

## Introduction

Cardiovascular disease (CVD) remains the leading cause of death globally.^1^ Because most atherosclerotic events are preventable through targeted management of modifiable risk factors, accurate and systematic risk assessment is a cornerstone of prevention.^2,3^ The 2021 European Society of Cardiology (ESC) Guidelines recommend Systematic Coronary Risk Estimation 2 (SCORE2) and SCORE2-OP to estimate 10-year risk of first-onset cardiovascular events and to guide treatment thresholds and shared decision-making.^3^

Despite the availability of validated tools, its implementation in routine care is inconsistent. Surveys indicate that fewer than half of clinicians regularly use formal calculators, with many relying on unaided clinical judgement, an approach linked to systematic underestimation of risk, particularly among higher-risk patients.^4–6^ Even when calculators are applied, treatment gaps persist, and a substantial proportion of high-risk individuals do not receive guideline-recommended treatments, contributing to avoidable morbidity and mortality.^4,7^

A key contributor to this implementation gap is the structure and usability of electronic health records (EHR).^8–10^ Variables required for risk estimation are often buried in free-text notes or dispersed across poorly integrated sections, creating burden and cognitive load at the point of care. Conventional natural language processing has shown that relevant information can be recovered from unstructured text, pointing to a practical route for workflow simplification.^11^

Large language models (LLMs) extend these capabilities with instruction-following and generative functions that support record summarization, clinical-note interpretation, and interactive assistance.^12–14^ However, evidence for safe, accurate clinical decision support remains mixed, with LLMs encoding broad clinical knowledge and performing strongly on benchmarks, but with variable and inconsistent outcomes on diagnostic reasoning.^15–17^ Within cardiovascular prevention specifically, applications are promising but remain insufficiently validated.^18^

The convergence of persistent gaps in CVD prevention, namely suboptimal risk stratification and limited EHR utility, with rapid advances in LLMs creates a timely opportunity to evaluate this system's role as an aid to guideline-based risk assessment. Accordingly, we benchmarked contemporary LLMs using simulated outpatient vignettes to assess (i) extraction of cardiovascular risk factors, including SCORE2 input variables from routine-style clinical text and (ii) generation of ESC-aligned risk categories, each compared against expert-adjudicated reference standards. Our main objective was to delineate foundational performance and limitations as a prerequisite for integrating LLMs into clinical decision support systems aimed at optimizing CVD prevention.

## Methods

### Study design

We conducted a prespecified, simulation-based evaluation of LLMs for cardiovascular risk stratification in accordance with the 2021 ESC prevention guidelines.^3^ The study used systematically developed clinical vignettes to benchmark model capabilities against a reference standard. No real patient data were used. The study protocol was previously registered on the Open Science Framework (https://doi.org/10.17605/OSF.IO/J2ZK9).

### Clinical vignette development and validation

Thirty clinical vignettes were developed by a senior cardiologist to emulate outpatient notes of patients undergoing cardiovascular risk stratification. Each vignette (100–200 words) included demographics, medical history, current medications, physical examination findings, laboratory results, and diagnostic investigations in free-text format. All vignettes incorporated SCORE2 input variables (age, sex, smoking status, systolic blood pressure, total cholesterol, HDL cholesterol) plus additional cardiovascular risk factors and modifiers. The set was designed to provide representation across sex, age strata (40–49, 50–59, 60–69 years), and ESC risk categories (low-to-moderate, high, very high). Ten vignettes represented SCORE2-ineligible conditions: atherosclerotic cardiovascular disease (ASCVD; *n* = 4), diabetes mellitus (*n* = 3), chronic kidney disease (CKD; *n* = 2), and familial hypercholesterolaemia (FH, *n* = 1).

Each vignette underwent evaluation by three cardiologists using a four-domain rubric (clinical relevance, completeness, realism, clarity) rated on a four-point Likert scale; unanimous ratings of 3–4 were required (I-CVI = 1.00). Vignettes not meeting this threshold were revised until full agreement.

Vignettes were developed in Portuguese and translated into English by the original author. English versions were reviewed by a native English-speaking cardiologist, with back-translation to confirm conceptual equivalence. Discrepancies were resolved by consensus.

### ESC cardiovascular risk stratification framework

Under the 2021 ESC Guidelines on cardiovascular disease prevention, patients are assigned to one of three final cardiovascular risk categories: Low-to-Moderate, High, or Very High.^3^ In adults aged 40–69 years without predefined high-risk conditions, risk is estimated using SCORE2 and categorized using age-specific thresholds. In contrast, patients with selected predefined conditions are assigned directly to a risk category without SCORE2 calculation. In this study, four such exception categories were evaluated: ASCVD, diabetes, CKD, and FH. The SCORE2 calibration for moderate-risk European countries, applicable to Portugal, was used for all calculations and age-specific threshold assignments. Accordingly, performance was assessed across three decision layers: extraction of relevant variables, determination of SCORE2 applicability, and assignment of the final ESC risk category.

### Models, deployment, and prompting

We evaluated a pragmatic sample of proprietary and open-source LLMs selected on the basis of local availability and broad contemporary use. The final set included eleven models (see Supplementary material online, *Table S1*).

All models were accessed through their native API or Azure platform using provider-default decoding settings, in inference-only mode and without fine-tuning on the study dataset. Each vignette was evaluated in a new, independent session to avoid cross-case memory effects.

A standardized prompt template was developed through iterative refinement, with pilot testing performed using Gemini-2.0-Flash and GPT-4o before the main benchmark (see Supplementary material online, *Tables S2* and *S3*). The final prompt instructed each model to extract cardiovascular risk factors in structured format, determine SCORE2 applicability, identify any exception precluding SCORE2 use, provide a numeric 10-year SCORE2 estimate when applicable, assign one of three final ESC risk categories, give a brief justification, and return a structured JSON output for downstream analysis.

The benchmark was designed as a zero-shot assessment of intrinsic guideline recall. Models were therefore required to retrieve and apply the relevant SCORE2 and exception logic from prior training rather than from in-context algorithmic instructions. Accordingly, the prompt did not embed the full SCORE2 algorithm, age-specific thresholds, or detailed exception rules. Prompt structure was identical in Portuguese and English, with only the vignette language adapted. Each model completed 60 evaluations: 30 Portuguese and 30 English. The final prompt template and pilot performance metrics are provided in the Supplementary material online, *Appendix S1* and Supplementary material online, *Tables S2*–*SS3*.

### Reference standard

A three-member Cardiovascular Risk Adjudication Committee, composed of senior cardiologists with recognized expertise in cardiovascular prevention and not involved in vignette development or model evaluation, independently evaluated each vignette across the same three decision layers used in the analytical framework: extraction of relevant cardiovascular risk factors and modifiers, determination of SCORE2 applicability, and assignment of the final ESC risk category. Initial disagreements in final ESC risk category assignment occurred in 3 of 30 vignettes (10%) and were resolved by structured discussion and consensus among all three committee members. The final adjudicated outputs constituted the reference (Gold Standard) against which LLMs were compared.

### Outcomes

The primary outcome was agreement with the reference standard for three-class ESC cardiovascular risk classification (Low-to-Moderate, High, Very High), measured using quadratic-weighted Cohen’s kappa (κw). Supplementary measures were overall accuracy and major error rate, defined as a two-class misclassification.

Secondary outcomes included: (i) risk-factor extraction accuracy for traditional cardiovascular risk factors listed in Supplementary material online, *Table S4*, assessed using micro- and macro-averaged precision, recall, and F1-scores, with per-vignette Jaccard similarity; (ii) risk-modifier extraction accuracy for the modifiers listed in Supplementary material online, *Table S4*, assessed using the same metrics; (iii) SCORE2 applicability, analysed as a binary decision problem (apply SCORE2 vs. withhold SCORE2) and further characterized by missed exceptions and false exceptions; (iv) numeric SCORE2 agreement in applicable cases, quantified using mean absolute error, Bland–Altman bias and limits of agreement, and Lin’s concordance correlation coefficient; and (v) language robustness, assessed by paired comparison of Portuguese and English outputs.

### Clinician comparator analysis

To contextualize LLM performance against human judgement, a pre-specified clinician comparator analysis was performed using eight physicians—three family medicine specialists, three internal medicine specialists, and two cardiologists, each with more than three years of clinical experience—who independently classified the same 30 Portuguese vignettes into the final three-class ESC cardiovascular risk categories.

Agreement between each clinician's ratings and the adjudicated Gold Standard was assessed using quadratic-weighted κw. The primary human comparator was defined as a pooled individual-level benchmark, in which all clinician ratings (8 raters × 30 vignettes = 240 ratings) were analysed jointly against the Gold Standard to estimate the level of agreement expected from an individual physician performing this task. Inter-rater agreement among clinicians was evaluated separately using weighted Gwet's AC2.

### Post hoc explanatory analyses in SCORE2-eligible vignettes

Two *post hoc* explanatory analyses were performed in SCORE2-eligible vignettes (*n* = 20). First, internal consistency was assessed by comparing each model’s reported final risk category with the category expected from its reported numeric SCORE2 estimate using ESC age-specific thresholds. Second, deterministic reconstruction was performed by calculating an external SCORE2 estimate from model-extracted variables using a spreadsheet-based implementation of the published ESC SCORE2 coefficients for moderate-risk countries and assigning the corresponding risk category using the same age-specific thresholds. Agreement with the Gold Standard was quantified using quadratic-weighted κw and compared with model-native κw in the same subset. These analyses were intended to distinguish downstream category-mapping errors from earlier errors in variable extraction or SCORE2 computation.

### Statistical analysis

All analyses were conducted in R (version 4.4.1) using RStudio (v2025.09.1 + 401; Posit Software, Boston, MA, USA). The Portuguese gold standard served as the reference for both languages. Each model generated 60 outputs (30 Portuguese and 30 English), paired by vignette identifier. Minor rounding/transcription differences in continuous variables were tolerated (±0.2). For risk-factor extraction, omitted model detections of gold-standard factors were treated as false negatives; vignettes with missing gold-standard values for a given factor were excluded from that analysis. For ESC risk classification, unclassifiable outputs were counted as incorrect for accuracy but excluded from ordinal agreement estimates; numeric comparisons were limited to vignettes with valid calculations from both model and reference.

For paired metrics (including quadratic-weighted kappa, accuracy, and safety-related error rates), 95% confidence intervals were obtained by non-parametric bootstrap resampling (2000 vignette-level replicates). Wilson score intervals were used for single proportions (e.g. sensitivity and specificity). Language differences were assessed using paired *t*-test or Wilcoxon signed-rank test for continuous outcomes and McNemar’s test for categorical outcomes, with Benjamini–Hochberg false discovery rate correction for multiple comparisons.

### Ethics

This study used synthetic clinical vignettes only; no real-patient data were included. For clinician benchmarking, eight physicians voluntarily rated vignettes and provided informed consent. The Luz Saúde Research and Ethics Committee (Lisbon, Portugal) determined the activity exempt from formal review (decision issued 4 June 2025). The project was coordinated by Hospital da Luz Learning Health.

## Results

### Vignette characteristics and gold-standard distribution

Thirty simulated outpatient clinical vignettes were evaluated (see Supplementary material online, *Appendix S2*), generating 60 assessments per model (30 Portuguese and 30 English). The vignettes represented a middle-aged population (mean age 54.6 ± 8.8 years, 50% male) with prevalent cardiovascular risk factors including hypertension (60%), dyslipidaemia (70%), and active smoking (37%) (*Table 1*). More than half (53%) included additional risk modifiers—one modifier in 23%, two in 20%, and three or more in 10% of cases. Twenty vignettes (66.7%) met standard SCORE2 eligibility criteria, while 10 (33.3%) involved predefined conditions requiring exception-based categorical risk assignment, including ASCVD (*n* = 4), diabetes mellitus (*n* = 3), CKD (*n* = 2), and familial hypercholesterolaemia (*n* = 1). Following expert adjudication, the gold-standard risk distribution comprised 5 (16.7%) low-to-moderate risk, 13 (43.3%) high risk, and 12 (40.0%) very-high risk vignettes, representing a higher-risk profile than initially expected (50%, 30%, and 20%, respectively) (see Supplementary material online, *Figures S1* and *S2*).

**Table 1:** Baseline clinical profile of simulated outpatient vignettes

Demographics	
Age, years^a^	54.6 ± 8.8 [36–67]
Male sex	15 (50.0%)
**Cardiovascular Risk Profile**	
Blood Pressure, mmHg	
Systolic blood pressure	135.3 ± 17.5 [107–189]
Diastolic blood pressure	79.9 ± 13.3 [58–107]
Lipids, mg/dL	
Total cholesterol	195.7 ± 45.3 [129–336]
HDL cholesterol	47.9 ± 9.1 [32–67]
Non-HDL cholesterol	147.8 ± 46.2 [79–219]
LDL cholesterol	119.1 ± 46.5 [49–258]
Triglycerides	137.8 ± 50.5 [62–256]
Risk factors	
Hypertension	18 (60.0%)
Dyslipidaemia^b^	21 (70.0%)
Smoking status^b^	
Current	11 (36.7%)
Former	7 (23.3%)
Never	10 (33.3%)
Predefined risk modifiers (any occurrence)	
Cancer	2 (6.7%)
Chronic inflammatory disease	1 (3.3%)
Chronic obstructive pulmonary disease	1 (3.3%)
Coronary calcium score = 0	1 (3.3%)
Elevated coronary calcium score	4 (13.3%)
Family history of premature ASCVD	1 (3.3%)
hs-CRP	1 (3.3%)
Increased arterial stiffness	1 (3.3%)
Lp(a) elevated	1 (3.3%)
Obesity	2 (6.7%)
Obstructive sleep apnoea	3 (10.0%)
Pre-diabetes	11 (36.7%)
Number of risk modifiers per vignette	
None	14 (46.7%)
One	7 (23.3%)
Two	6 (20.0%)
Three or more	3 (10.0%)
**SCORE2 applicability**	
Eligible (no exception)	20 (66.7%)
SCORE2 exceptions for risk stratification	10 (33.3%)
Prior ASCVD	4 (13.3%)
Diabetes	3 (10.0%)
Chronic kidney disease	2 (6.7%)
Familial hypercholesterolemia	1 (3.3%)
**ESC risk category**	
Low-to-Moderate	5 (16.7%)
High	13 (43.3%)
Very High	12 (40.0%)

**Data presentation:** Values are reported as mean ± standard deviation [range] for continuous variables and *n* (%) for categorical variables. Percentages are calculated based on *n* = 30 vignettes unless otherwise noted. **Definitions:** ‘SCORE2 applicability’ distinguishes vignettes eligible for standard calculation from those requiring guideline-based exceptions.

^a^Age includes one 36-year-old patient with familial hypercholesterolemia; all other vignettes represent patients aged ≥40 years.

^b^Denotes variables with missing data in the source vignettes (unknown dyslipidaemia: *n* = 2; unknown smoking status: *n* = 2). **Abbreviations:** ASCVD, atherosclerotic cardiovascular disease; HDL, high-density lipoprotein; LDL, low-density lipoprotein; Lp(a), lipoprotein(a); hs-CRP, high-sensitivity C-reactive protein.

### Performance benchmarks: risk factor and modifier extraction

**Figure 1:**
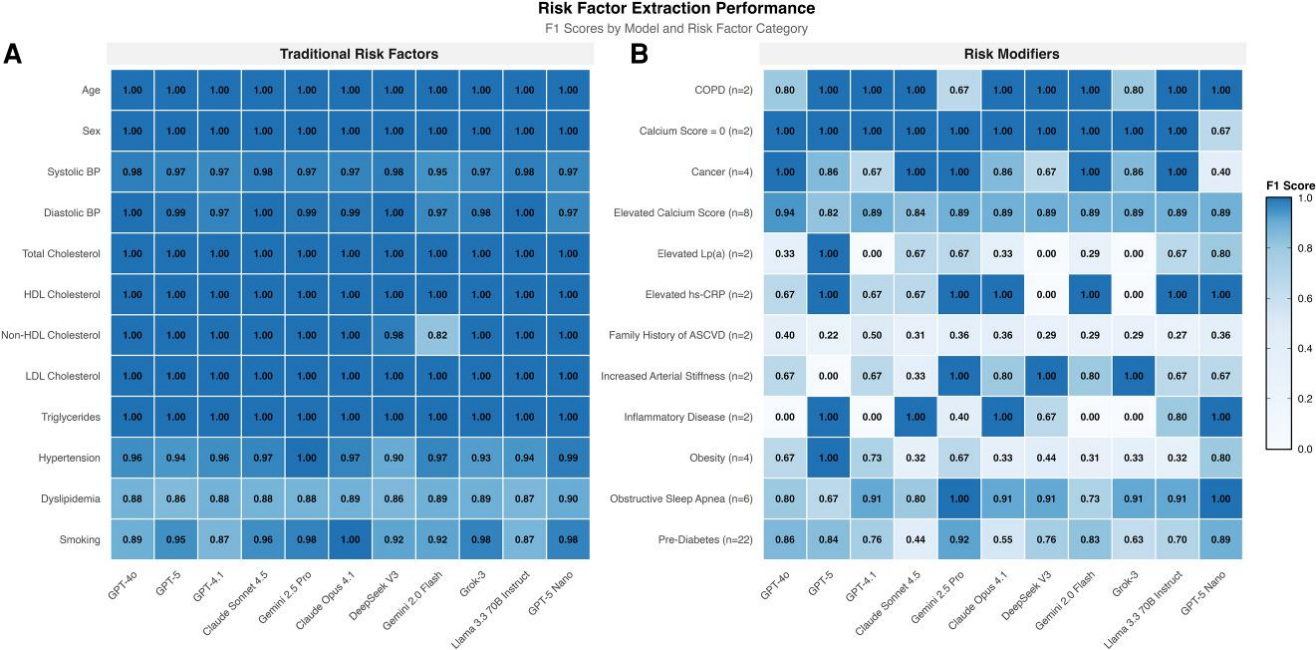
Extraction accuracy for traditional cardiovascular risk factors and risk modifiers by model. Extraction performance heatmaps. (*A*) Per-factor F1-scores for the 12 traditional cardiovascular risk factors. (*B*) Per-factor F1-scores for the 12 predefined risk modifiers; the prevalence of each modifier in the reference dataset (*N*) is provided in parentheses. **Data:** Values represent pooled F1-scores (harmonic mean of precision and recall) across Portuguese and English vignettes (N = 60 per model); Models are ordered from left to right by decreasing κw performance. **Abbreviations:** BP, Blood Pressure; COPD, chronic obstructive pulmonary disease; Lp(a), lipoprotein(a); hs-CRP, high-sensitivity C-reactive protein; ASCVD, atherosclerotic cardiovascular disease.

All eleven models demonstrated excellent performance in extracting traditional cardiovascular risk factors, with micro-averaged F1 scores ranging from 0.97 to 0.99 (*Table 2*, Supplementary material online, *Figures S3*-*S4*). Extraction of the six SCORE2 input variables was uniformly high across all models (micro-F1: 0.98–0.99), with near-perfect performance for age, sex, and lipid parameters. Smoking status showed the greatest variability among SCORE2 inputs (F1: 0.94, 95% CI: 0.91–0.96). Among additional traditional factors, dyslipidaemia diagnosis showed the lowest extraction accuracy (F1: 0.88, 95% CI: 0.86–0.90), while continuous variables were extracted with near-perfect reliability (*Figure 1*, Supplementary material online, *Tables S5*-*S7*).

**Table 2:** Extraction performance by model: cardiovascular risk factors, SCORE2 core risk factors, and risk modifiers

Model	Cardiovascular risk factors					SCORE2 input risk factors	Risk modifiers
	Micro-F1	Micro-P	Micro-R	Macro-F1	Jaccard	Micro-F1	Micro-F1
GPT-4o	0.98 (0.98–0.99)	0.98 (0.96–0.99)	0.99 (0.98–0.99)	0.98 (0.95–1.00)	0.97 (0.95–0.98)	0.99 (0.98–1.00)	0.74 (0.61–0.84)
GPT-5	0.98 (0.97–0.99)	0.99 (0.98–1.00)	0.97 (0.96–0.98)	0.98 (0.95–0.99)	0.97 (0.95–0.98)	0.98 (0.97–0.99)	0.77 (0.66–0.87)
GPT-4.1	0.98 (0.97–0.99)	0.98 (0.97–0.99)	0.97 (0.96–0.98)	0.97 (0.94–0.99)	0.96 (0.94–0.97)	0.99 (0.98–0.99)	0.80 (0.68–0.90)
Claude Sonnet 4.5	0.99 (0.98–0.99)	0.98 (0.97–0.99)	0.99 (0.98–1.00)	0.98 (0.96–1.00)	0.97 (0.96–0.98)	0.99 (0.99–1.00)	0.58 (0.45–0.68)
Gemini 2.5 Pro	0.99 (0.98–0.99)	0.98 (0.97–0.99)	0.99 (0.98–1.00)	0.99 (0.96–1.00)	0.98 (0.96–0.99)	0.99 (0.98–0.99)	0.81 (0.70–0.90)
Claude Opus 4.1	0.99 (0.98–0.99)	0.99 (0.97–0.99)	0.99 (0.98–0.99)	0.99 (0.96–1.00)	0.98 (0.96–0.99)	0.99 (0.98–0.99)	0.64 (0.47–0.77)
DeepSeek V3	0.98 (0.97–0.98)	0.97 (0.95–0.98)	0.99 (0.98–0.99)	0.97 (0.94–1.00)	0.96 (0.94–0.97)	0.99 (0.98–0.99)	0.67 (0.52–0.78)
Gemini 2.0 Flash	0.97 (0.95–0.97)	0.98 (0.96–0.99)	0.95 (0.93–0.97)	0.96 (0.93–0.99)	0.93 (0.92–0.95)	0.99 (0.98–0.99)	0.67 (0.54–0.77)
Grok-3	0.98 (0.97–0.99)	0.98 (0.97–0.99)	0.98 (0.97–0.99)	0.98 (0.96–1.00)	0.97 (0.95–0.98)	0.98 (0.97–0.99)	0.60 (0.43–0.73)
Llama 3.3 70B Instruct	0.98 (0.97–0.99)	0.98 (0.97–0.99)	0.98 (0.97–0.99)	0.97 (0.94–1.00)	0.96 (0.95–0.98)	0.98 (0.97–0.99)	0.67 (0.52–0.78)
GPT-5 Nano	0.99 (0.98–0.99)	0.98 (0.97–0.99)	0.99 (0.97–0.99)	0.98 (0.96–1.00)	0.97 (0.96–0.98)	0.99 (0.98–1.00)	0.82 (0.70–0.90)

**Metrics:** Data represent micro-averaged F1-scores, precision, and recall, macro-averaged F1-scores, and Jaccard similarity coefficients, presented with 95% confidence intervals in parentheses. **Sample size:** Each model evaluation includes *n* = 60 predictions (pooled 30 Portuguese and 30 English). **Definitions:** ‘Cardiovascular Risk Factors’ comprises the 12-item traditional panel (6 SCORE2 core inputs risk factors + 6 additional factors); ‘Risk Modifiers’ comprises the 12 predefined guideline modifiers; Models are ordered by quadratic-weighted kappa (κw) from the primary three-class risk analysis. **Abbreviations:** CI, confidence interval; SCORE2, Systematic Coronary Risk Estimation 2.

Risk modifier extraction was substantially more challenging, with micro-F1 scores ranging from 0.58 to 0.82 across models—markedly lower and more variable than traditional factor performance (*Table 2* and Supplementary material online, *Table S8*). The most consistently difficult modifiers were family history of premature ASCVD (F1: 0.32, 95% CI: 0.21–0.42), elevated lipoprotein(a) (F1: 0.43, 95% CI: 0.26–0.60), and obesity (F1: 0.45, 95% CI: 0.36–0.53) (*Figure 1*, Supplementary material online, *Table S9*).

### Primary outcome: ESC three-class risk classification

Of the eleven evaluated models, eight returned complete classifications for all 60 vignettes. GPT-5 withheld classification in four vignettes where smoking status was absent; Llama 3.3 70B Instruct and GPT-5 Nano omitted classifications for one and five vignettes, respectively, without explanation.

**Figure 2:**
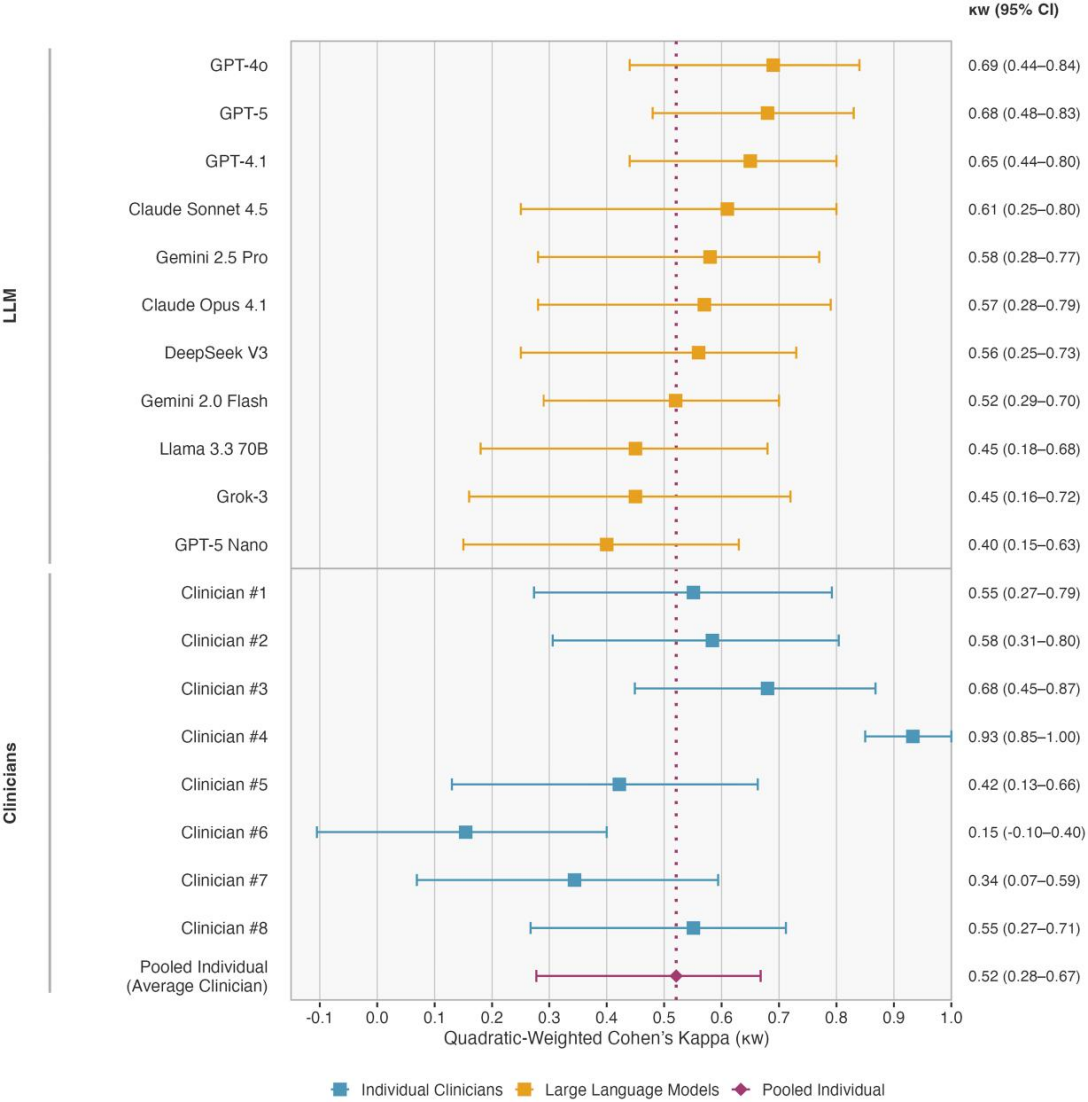
**Agreement of LLMs and clinicians with the adjudicated gold standard for three-class ESC cardiovascular risk classification.** Forest plot of agreement with the adjudicated Gold Standard for final three-class ESC cardiovascular risk classification, shown as quadratic-weighted Cohen’s kappa (κw) with 95% confidence intervals. The pooled individual-level clinician benchmark combines all 240 clinician ratings (8 clinicians × 30 vignettes); the vertical dotted line marks this benchmark (κw = 0.52).

Model performance varied substantially (*Table 3*, *Figure 2*). GPT-4o achieved the highest concordance with the gold standard (κw = 0.69, 95% CI: 0.44–0.84), followed by GPT-5 (κw = 0.68, 95% CI: 0.48–0.83) and GPT-4.1 (κw = 0.65, 95% CI: 0.44–0.80), all demonstrating moderate to substantial agreement. GPT-5 Nano showed the lowest concordance (κw = 0.40, 95% CI: 0.15–0.63). Overall classification accuracy ranged from 67% (GPT-4o) to 42% (GPT-5 Nano).

**Table 3:** Three-class ESC risk classification: model performance summary (ordered by κw)

Model	N/Unknown (%)	Agreement κw (95% CI)	Accuracy (95% CI)	Errors			
				Minor (%)	Major (%)	Over (%)	Under (%)
**GPT-4o**	60/0%	0.69 (0.44–0.84)	0.67 (0.52–0.82)	33.3	0.0	8.3	25.0
**GPT-5**	56/6.7%	0.68 (0.48–0.83)	0.58 (0.42–0.75)	37.5	0.0	3.6	33.9
**GPT-4.1**	60/0%	0.65 (0.44–0.80)	0.62 (0.45–0.77)	38.3	0.0	3.3	35.0
**Claude Sonnet 4.5**	60/0%	0.61 (0.25–0.80)	0.63 (0.45–0.78)	35.0	1.7	26.7	10.0
**Gemini 2.5 Pro**	60/0%	0.58 (0.28–0.77)	0.57 (0.42–0.73)	43.3	0.0	20.0	23.3
**Claude Opus 4.1**	60/0%	0.57 (0.28–0.79)	0.60 (0.43–0.77)	36.7	3.3	10.0	30.0
**DeepSeek V3**	60/0%	0.56 (0.25–0.73)	0.52 (0.35–0.67)	48.3	0.0	18.3	30.0
**Gemini 2.0 Flash**	60/0%	0.52 (0.29–0.70)	0.55 (0.40–0.70)	41.7	3.3	5.0	40.0
**Grok-3**	60/0%	0.45 (0.16–0.72)	0.53 (0.37–0.70)	40.0	6.7	10.0	36.7
**Llama 3.3 70B Instruct**	59/1.7%	0.45 (0.18–0.68)	0.58 (0.42–0.73)	32.2	8.5	3.4	37.3
**GPT-5 Nano**	55/8.3%	0.40 (0.15–0.63)	0.42 (0.25–0.58)	41.8	12.7	7.3	47.3

**Outcomes:** ‘Agreement (κw)’ and ‘Errors’ are calculated using valid ordinal predictions only; ‘Accuracy’ is calculated against the total *n*, penalizing unclassifiable responses. **Definitions:** ‘N’ denotes the total evaluated predictions (30 Portuguese + 30 English); ‘Unknown (%)’ represents the proportion of vignettes where the model failed to return a classification or output ‘unknown.’; ‘Minor Error’ indicates misclassification by one adjacent category; ‘Major Error’ indicates misclassification by two categories (Low-to-Moderate ↔ Very High); ‘Over/Under’ refers to the direction of minor errors; Models are ordered by quadratic-weighted kappa (κw) from the primary three-class risk analysis. **Abbreviations:** CI, confidence interval; ESC, European Society of Cardiology; κw, quadratic-weighted kappa.

**Figure 3:**
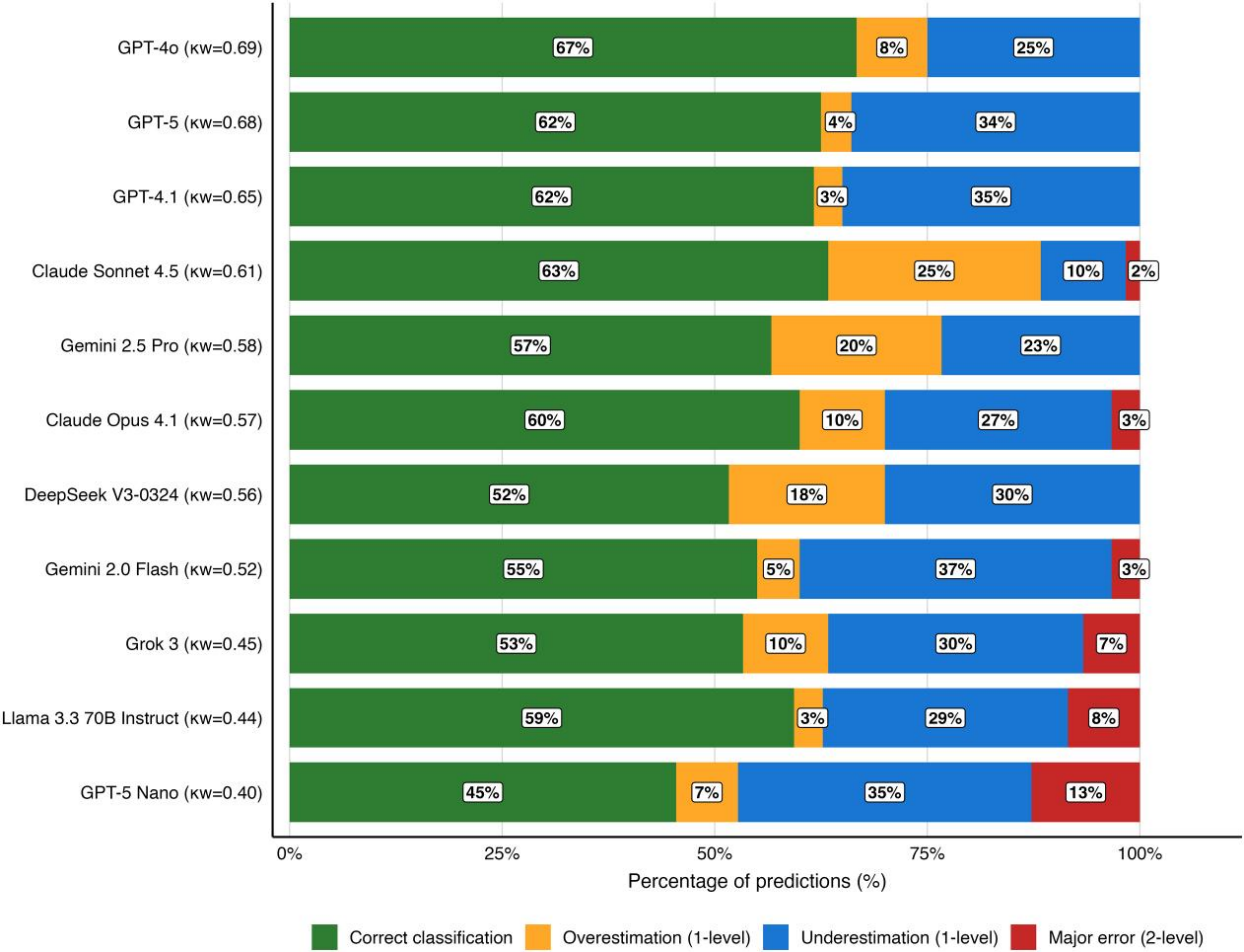
**Model-level accuracy and error direction in three-class ESC risk classification.** Distribution of risk classification errors. Stacked bars display the proportion of predictions classified as Correct (green), Minor Overestimation (1-level, orange), Minor Underestimation (1-level, blue), or Major Error (2-level, red). **Data:** Percentages are calculated relative to valid predictions; unclassifiable (‘unknown’) outputs are excluded from the visualization. Models are ordered by quadratic-weighted kappa (κw) values, shown in parentheses. Results are pooled across languages. **Abbreviations:** ESC, European Society of Cardiology.

Analysis of error patterns revealed clinically relevant safety signals (*Figures 3*, Supplementary material online, *Figure S5*). Major two-category errors were rare but present in six models, with GPT-5 Nano showing the highest rate (13%); five models (GPT-4o, GPT-5, GPT-4.1, Gemini 2.5 Pro, DeepSeek V3) achieved zero major errors. Ten of eleven models systematically underestimated risk, with the sole exception being Claude Sonnet 4.5, which overestimated more frequently. GPT-4o achieved the best balance of sensitivity and specificity for high and very high-risk detection (92% and 100% respectively), while Claude Sonnet 4.5 reached perfect sensitivity (100%) at the cost of reduced specificity (80%) (see Supplementary material online, *Table S10*). The predominance of underestimation is clinically relevant because it may shift patients away from higher-risk categories that trigger more intensive preventive management under ESC guidance.

### SCORE2 applicability decisions

**Figure 4:**
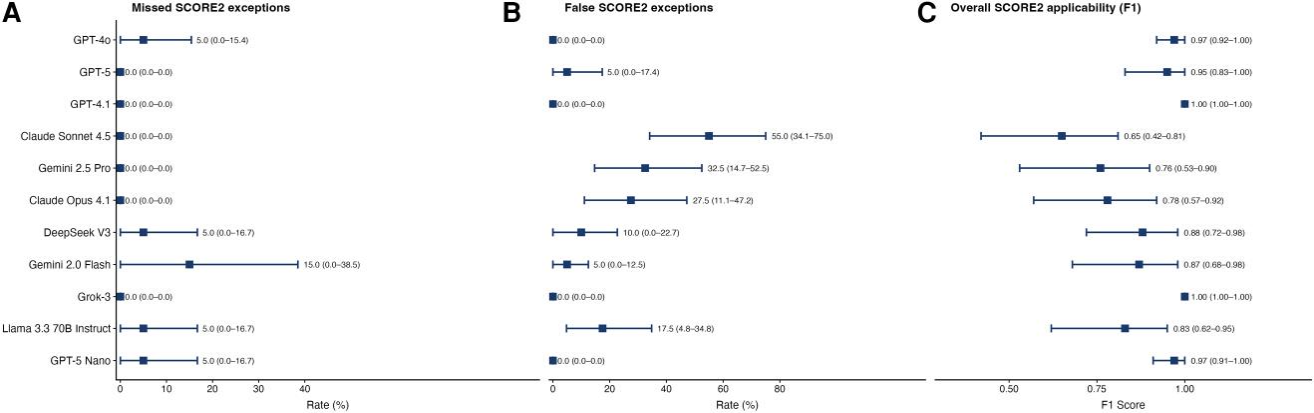
**SCORE2 applicability errors and overall performance. (** *A* **)** Missed SCORE2 exceptions: rate at which models incorrectly applied SCORE2 to patients requiring a guideline-based exception (safety error; lower is better). **(***B***)** False SCORE2 exceptions: rate at which models incorrectly withheld SCORE2 from eligible patients (usability error; lower is better). **(***C***)** Overall SCORE2 applicability (F1): binary classification performance for the eligibility decision, balancing detection of true exceptions against avoidance of false ones (higher is better). **Data:** Squares represent point estimates; horizontal lines represent 95% bootstrap confidence intervals. Point estimates and 95% CIs are displayed to the right of each bar. Models are ordered by quadratic-weighted κ from the primary three-class ESC risk classification analysis. **Abbreviations:** CI, confidence interval; F1, F1-score; SCORE2, Systematic Coronary Risk Estimation 2.

Models showed variable performance in determining whether SCORE2 should be applied or replaced by exception-based risk assignment (*Figure 4*, Supplementary material online, *Table S11*). GPT-4.1 and Grok-3 achieved perfect applicability classification (F1 = 1.00), whereas Claude Sonnet 4.5 performed worst (F1 = 0.65, 95% CI: 0.42–0.81). Missed exceptions were uncommon for all models except with Gemini 2.0 Flash (15.0%, 95% CI: 0.0–38.5%). Variation was driven mainly by false exceptions, ranging from 0% in four models (GPT-4o, GPT-4.1, Grok-3, GPT-5 Nano) to 55.0% (95% CI: 34.1–75.0) for Claude Sonnet 4.5, with Gemini 2.5 Pro (32.5%, 95% CI: 14.7–52.5) and Claude Opus 4.1 (27.5%, 95% CI: 11.1–47.2) also notably elevated. When SCORE2 was erroneously withheld, five models consistently cited only guideline-specified contraindications (GPT-4o, GPT-5, GPT-4.1, Grok-3, GPT-5 Nano; 100% valid), whereas the remaining six invoked non-qualifying conditions in a proportion of cases. From a workflow perspective, false exception assignment reduces usability by unnecessarily diverting otherwise eligible patients away from standard SCORE2-based assessment.

### Numeric SCORE2 accuracy

Among vignettes where SCORE2 risk calculation was applicable, numeric agreement between model-predicted 10-year cardiovascular risk and the Gold Standard varied substantially across models (*Table 4*, Supplementary material online, *Table S12*, Supplementary material online, *Figure S6*). Only three models (GPT-4o, GPT-4.1, and Grok-3) produced numeric SCORE2 estimates for all 40 eligible outputs; the remaining models generated numeric estimates for 18 to 38 of 40 eligible outputs, indicating variable completeness of numeric reporting across models (*Table 4*). Mean absolute error (MAE) ranged from 1.22 percentage points for Claude Sonnet 4.5 (95% CI, 0.53–2.29; *n* = 18) to 3.16 for Gemini 2.0 Flash (95% CI, 2.44–3.87; *n* = 38), with all models exceeding one percentage point on average. Bland–Altman analyses showed a tendency towards underestimation in all models except GPT-4o, whose bias estimate crossed zero (bias, −0.89; 95% CI, −1.89 to 0.11). Lin’s concordance correlation coefficients ranged from 0.43 to 0.83, indicating at best moderate agreement with reference SCORE2 values.

**Table 4:** Numeric agreement with SCORE2 (applicable cases only)

Model	*n*	MAE (95% CI)	Bias (95% CI)	CCC (95% CI)
**GPT-4o**	40	2.27 (1.53, 3.04)	−0.89 (−1.89, 0.11)	0.65 (0.44, 0.79)
**GPT-5**	33	1.90 (1.22, 2.74)	−1.56 (−2.48, −0.80)	0.71 (0.42, 0.89)
**GPT-4.1**	40	2.29 (1.70, 2.93)	−1.69 (−2.58, −0.76)	0.66 (0.45, 0.80)
**Claude Sonnet 4.5**	18	1.22 (0.53, 2.29)	−0.97 (−2.12, −0.24)	0.83 (0.48, 0.97)
**Gemini 2.5 Pro**	27	1.29 (0.69, 2.15)	−1.05 (−1.97, −0.38)	0.79 (0.51, 0.94)
**Claude Opus 4.1**	29	1.67 (0.91, 2.68)	−1.31 (−2.44, −0.46)	0.65 (0.28, 0.90)
**DeepSeek V3**	36	2.38 (1.52, 3.29)	−2.20 (−3.12, −1.29)	0.51 (0.27, 0.73)
**Gemini 2.0 Flash**	38	3.16 (2.44, 3.87)	−3.11 (−3.84, −2.34)	0.43 (0.28, 0.54)
**Grok-3**	40	1.92 (1.26, 2.63)	−1.49 (−2.34, −0.65)	0.59 (0.31, 0.78)
**Llama 3.3 70B Instruct**	31	2.68 (1.98, 3.43)	−1.93 (−3.02, −0.77)	0.56 (0.32, 0.73)
**GPT-5 Nano**	31	2.23 (1.64, 2.84)	−2.07 (−2.73, −1.40)	0.59 (0.41, 0.71)

**Sample determination:** Analysis is restricted to the subset of vignettes (‘N’) where both the Gold Standard was applicable, and the model successfully generated a numeric risk value. **Metrics:** Values represent percentage points of 10-year cardiovascular risk; ‘Bias’ is calculated as Mean(Model) − Mean(Gold Standard), where positive values indicate overestimation; ‘CCC’ denotes Lin’s Concordance Correlation Coefficient (−1 to 1); Models are ordered by κw from the primary risk analysis. **Abbreviations:** CI, confidence interval; MAE, mean absolute error; SCORE2, Systematic Coronary Risk Estimation 2.

### Internal consistency and deterministic risk recalculation

In the *post hoc* analysis, internal consistency between models reported numeric SCORE2 estimates and their stated final risk categories was variable across the eleven LLMs (*Table 5*). Numeric-category agreement ranged from 37.5% (Claude Sonnet 4.5) to 85.0% (Gemini 2.0 Flash), indicating that a meaningful proportion of outputs contained internally contradictory reasoning—models produced a numeric estimate but then assigned a category inconsistent with that figure under ESC age-specific thresholds. Original κw in this SCORE2-eligible subset ranged from 0.15 (GPT-5 Nano) to 0.61 (GPT-4o), broadly consistent with performance in the full vignette set.

**Table 5:** Post hoc analyses of internal consistency and deterministic risk recalculation in SCORE2-eligible vignettes

	Internal consistency analysis		Recalculated risk category analysis		
Model	Evaluable outputs, *n*	Numeric-category agreement, *n* (%)	Original κw (95% CI)	Deterministic κw (95% CI)	Δκw (95% CI)
**GPT-4o**	40	30 (75.0%)	0.61 (0.38–0.75)	0.90 (0.70–1.00)	+0.29 (0.13–0.49)
**GPT-5**	35	30 (85.7%)	0.57 (0.35–0.72)	0.87 (0.66–1.00)	+0.30 (0.08–0.54)
**GPT-4.1**	40	31 (77.5%)	0.52 (0.31–0.66)	0.87 (0.66–1.00)	+0.35 (0.19–0.55)
**Claude Sonnet 4.5**	40	15 (37.5%)	0.60 (0.20–0.82)	0.90 (0.70–1.00)	+0.30 (0.01–0.71)
**Gemini 2.5 Pro**	40	25 (62.5%)	0.44 (0.06–0.63)	0.90 (0.70–1.00)	+0.46 (0.22–0.81)
**Claude Opus 4.1**	40	21 (52.5%)	0.46 (0.17–0.71)	0.90 (0.70–1.00)	+0.44 (0.17–0.73)
**DeepSeek V3**	40	32 (80.0%)	0.48 (0.21–0.63)	0.90 (0.70–1.00)	+0.42 (0.24–0.67)
**Gemini 2.0 Flash**	40	34 (85.0%)	0.45 (0.23–0.61)	0.90 (0.70–1.00)	+0.45 (0.24–0.69)
**Grok-3**	40	27 (67.5%)	0.27 (0.02–0.55)	0.87 (0.66–1.00)	+0.60 (0.29–0.88)
**Llama 3.3 70B Instruct**	38	22 (57.9%)	0.32 (0.07–0.58)	0.85 (0.63–0.97)	+0.53 (0.21–0.80)
**GPT-5 Nano**	31	18 (58.1%)	0.15 (0.04–0.30)	0.87 (0.66–1.00)	+0.72 (0.44–0.93)

Analyses were restricted to SCORE2-eligible vignettes (*n* = 20; up to 40 pooled outputs per model across both languages). **Internal consistency analysis:** ‘Evaluable outputs’ denotes responses in which the model judged SCORE2 applicable and provided both a numeric SCORE2 estimate and a final risk category. Numeric-category agreement indicates concordance between the reported final category and that expected from the model’s own numeric SCORE2 estimate using ESC age-specific thresholds for moderate-risk countries and is reported as *n* (%) using evaluable outputs as the denominator. **Recalculated risk category analysis:** Original κw denotes agreement with the Gold Standard recalculated within the same SCORE2-eligible subset; deterministic κw reflects agreement after rule-based reassignment of final category from model-extracted variables; Δκw = deterministic κw − original κw. **Abbreviations:** CI, confidence interval; κw, quadratic-weighted Cohen’s kappa.

Deterministic risk recalculation yielded substantially and consistently higher agreement with the Gold Standard across all models (*Table 5*). Deterministic κw clustered narrowly between 0.85 and 0.90, and all eleven models showed a positive Δκw—with confidence intervals excluding zero in nine of eleven models. The magnitude of improvement was largest for GPT-5 Nano (Δκw = + 0.72, 95% CI: 0.44–0.93), Grok-3 (+0.60, 95% CI: 0.29–0.88), and Llama 3.3 70B Instruct (+0.53, 95% CI: 0.21–0.80), models with the lowest original κw, and smallest for GPT-4o (+0.29, 95% CI: 0.13–0.49), which had the highest native performance at baseline. The marked improvement after deterministic risk categorization indicates that much of the observed classification error arose downstream of variable extraction, particularly during numeric computation, rule application, or final category mapping.

### Bilingual performance: Portuguese vs English

Performance was generally consistent across Portuguese and English vignettes (*Table 6*, Supplementary material online, *Figure S7*). For risk factor extraction, Micro-F1 exceeded 0.95 in both languages for all models, with only minimal between-language differences (ΔMicro-F1, −0.01 to 0.02). Risk classification showed greater variability, with the largest differences observed for Llama 3.3 70B Instruct (Δκw = 0.22, 95% CI −0.04 to 0.49; better in English) and Gemini 2.0 Flash (Δκw = −0.16, 95% CI −0.38 to 0.07; better in Portuguese). SCORE2 applicability classification was also stable across languages, with ΔF1 ranging from −0.05 to 0.09; the largest absolute difference was observed for Llama 3.3 70B Instruct (ΔF1 = 0.09, 95% CI −0.06 to 0.27). No statistically significant language effects were identified after FDR correction.

**Table 6:** Language performance across portuguese and English vignettes

Model	Risk factors (Micro-F1)			ESC risk class (κw)			SCORE2 applicability (F1)		
	PT	EN	ΔF1 [95% CI]	PT	EN	Δκw [95% CI]	PT	EN	ΔF1 [95% CI]
**GPT-4o**	0.99	0.98	−0.01 (−0.01–0.00)	0.69	0.68	−0.01 (−0.12–0.09)	1.00	0.95	−0.05 (−0.20–0.00)
**GPT-5**	0.97	0.98	0.01 (0.00–0.02)	0.68	0.69	0.02 (−0.10–0.13)	0.95	0.95	+0.00 (0.00–0.00)
**GPT-4.1**	0.98	0.98	−0.00 (−0.01–0.01)	0.60	0.69	0.09 (−0.02–0.23)	1.00	1.00	+0.00 (−0.06–0.06)
**Claude Sonnet 4.5**	0.98	0.99	0.00 (−0.00–0.01)	0.59	0.62	0.04 (−0.17–0.27)	0.65	0.65	+0.03 (−0.07–0.13)
**Gemini 2.5 Pro**	0.98	0.98	−0.00 (−0.01–0.01)	0.64	0.53	−0.10 (−0.24–0.00)	0.74	0.77	+0.03 (−0.08–0.15)
**Claude Opus 4.1**	0.98	0.98	−0.00 (−0.01–0.00)	0.63	0.52	−0.11 (−0.28–0.07)	0.77	0.80	−0.03 (−0.20–0.13)
**DeepSeek V3**	0.98	0.98	0.00 (−0.00–0.01)	0.60	0.53	−0.07 (−0.21–0.06)	0.90	0.87	+0.03 (−0.15–0.22)
**Gemini 2.0 Flash**	0.96	0.98	0.02 (0.01–0.03)	0.61	0.45	−0.16 (−0.38–0.07)	0.86	0.89	+0.03 (−0.15–0.22)
**Grok-3**	0.98	0.98	−0.00 (−0.01–0.01)	0.44	0.46	0.02 (−0.07–0.15)	1.00	1.00	+0.00 (0.00–0.00)
**Llama 3.3 70B Instruct**	0.98	0.97	−0.01 (−0.02–0.00)	0.34	0.56	0.22 (−0.04–0.49)	0.78	0.87	+0.09 (−0.06–0.27)
**GPT-5 Nano**	0.99	0.98	−0.01 (−0.02–0.00)	0.33	0.46	0.13 (−0.08–0.36)	0.95	1.00	+0.05 (0.00–0.20)

Paired performance analysis between Portuguese (PT) and English (EN) vignettes across three domains: traditional risk factor extraction (Micro-F1), ESC risk classification (κw), and SCORE2 applicability (F1). Δ represents the difference (EN − PT); values are absolute differences with 95% confidence intervals shown in parentheses. No statistically significant differences were found after FDR correction. Models are ordered by κw. Abbreviations: CI, confidence interval; κw, quadratic-weighted kappa.

### Contextualizing LLM performance against physician benchmarks

Eight clinicians independently classified the 30 Portuguese vignettes using the ESC three-class cardiovascular risk system. Agreement with the adjudicated Gold Standard varied markedly across raters, with quadratic-weighted Cohen's κ ranging from 0.15 to 0.93, indicating substantial heterogeneity in individual physician judgement (*Figure 2*). No clinician produced a major two-class misclassification, contrary to what occurred in several LLM outputs. The prespecified: benchmark yielded a quadratic-weighted κ of 0.52 (95% CI, 0.28 to 0.67), representing moderate agreement with the reference standard. Notably, five of eleven LLMs equalled or exceeded this pooled clinician benchmark, suggesting that top-performing models reach the performance level of the average individual physician on this task. Inter-rater agreement among clinicians, reflecting the intrinsic reproducibility of human judgement was moderate (AC2 = 0.44, 95% CI: 0.27 to 0.55).

## Discussion

In this evaluation of eleven contemporary LLMs for cardiovascular risk stratification according to the ESC framework, performance separated clearly across the three decision layers of the task.^3^ Models achieved near-perfect extraction of traditional cardiovascular risk factors from clinical text, whereas the final three-class ESC risk classification remained only moderate and variable across systems, with GPT-4o achieving the highest agreement (κw = 0.69). SCORE2 applicability decisions were comparatively more reliable, particularly from a safety perspective, with few missed exception cases in most models, although false exception assignment substantially reduced usability in some systems. Numeric SCORE2 estimation was less robust: completion was inconsistent across models, mean absolute error exceeded one percentage point in all cases, and concordance with reference values was generally only moderate. Most importantly, *post hoc* deterministic recalculation from model-extracted variables substantially improved agreement with the Gold Standard across all models, with recalculated κw values clustering between 0.85 and 0.90. Taken together, these findings suggest that current LLMs are already strong at extracting relevant structured information from unstructured clinical notes, but that their main limitations in this setting arise downstream, during formal risk computation, exception handling, and final category assignment rather than in identification of the core input variables.

The marked gap between extraction performance and final ESC risk classification is not unexpected, because these tasks differ substantially in cognitive complexity. Extraction of traditional cardiovascular risk factors is largely a recognition task based on explicit and usually well-structured information in clinical text, which helps explain the near-perfect identification of age, blood pressure, and lipid values across models.^11,19–22^ By contrast, performance declined slightly for variables requiring interpretive judgement rather than simple retrieval, such as smoking status, hypertension, or dyslipidaemia labels. This difficulty became more evident for risk modifiers, many of which depend on contextual interpretation, implicit wording, or less standardized definitions, and were therefore detected less consistently.^21,22^

Establishing a final ESC risk classification is a sequential reasoning task rather than a simple extraction problem. It requires identification of the relevant variables, determination of SCORE2 applicability, recognition of exception-based conditions, correct risk estimation, and assignment of the final age-specific category according to the ESC framework.^3^ Errors may therefore emerge even when the core input variables are correctly identified. This interpretation is consistent with wider evidence showing that LLMs often perform well in information retrieval and structured summarization, but less reliably in multi-step clinical inference, requiring rule application and integration of multiple competing factors into a final judgement.^16,23,24^

The *post hoc* analyses help clarify where performance deteriorated. Despite high-fidelity extraction of the structured inputs required for SCORE2, several models did not translate this information into coherent downstream risk adjudication. Internal consistency between model-reported numeric SCORE2 values and their own final categorical assignments was variable, indicating that some errors arose not from failure to identify the relevant inputs, but from later stages of numeric computation or mapping to the correct age-specific ESC risk category. Clinically, this distinction matters, because a model may appear to interpret the case correctly while still producing a discordant final classification.

This interpretation was strengthened by deterministic recalculation. When SCORE2 was recomputed externally from model-extracted variables, agreement with the Gold Standard improved substantially across all systems, and recalculated κw values clustered in a high range despite the more modest agreement of native model outputs. These findings suggest that the main bottleneck in this setting lay downstream of extraction, in formal risk calculation and category assignment rather than in recognition of the core clinical data. From an implementation perspective, these results support a modular workflow in which LLMs assist with extraction and structuring of information from free-text notes, whereas numeric risk computation and final categorization are performed by deterministic calculators or guideline-based algorithms.^24–26^

An additional point of clinical relevance is the directional pattern observed in numeric estimation. Most models tended towards underestimation relative to the reference standard, which is potentially concerning in preventive cardiology because systematic downward bias may delay intensification of treatment in patients at highest need. This pattern is also consistent with prior literature showing that unaided physician judgement frequently underestimates cardiovascular risk, particularly in higher-risk individuals.^6,27^ However, the present study cannot determine the origin of this bias.^28,29^

SCORE2 applicability performance was generally more reassuring than final risk categorization, particularly from a safety perspective. In most models, missed exceptions were uncommon, suggesting some capacity to recognize when SCORE2 should be withheld in favour of direct guideline-based categorization.^3^ Clinically, this matters because failure to detect such cases may lead to inappropriate reliance on numeric risk estimation when categorical assignment is required. However, this relative strength was offset in several models by frequent false exception assignment. Although such errors are less likely to cause risk underestimation, they may substantially reduce usability by unnecessarily withholding SCORE2 in patients who were otherwise eligible for standard assessment.

This pattern highlights an important distinction between safety and operational usefulness. A model that over-identifies exceptions may appear cautious, yet still depart from the intended ESC framework and generate outputs that are difficult to trust when the underlying rationale is incorrect. In this study, false exception assignment was not a marginal issue, but a recurring source of performance loss in several systems. Taken together, these findings suggest that current LLMs may often recognize that a case is clinically complex, while still lacking the precision required to apply exception rules reliably enough for autonomous use. This again supports a supervised, assistive role for LLMs within cardiovascular prevention workflows rather than standalone end-to-end risk adjudication.^16,24,25^

The clinician benchmark provides important context for interpreting these findings. Agreement with the Gold Standard varied substantially across individual physicians, indicating that ESC-based cardiovascular risk classification was not a trivial task even for practicing clinicians. In that context, several LLMs achieved agreement within, or above, the range expected from an individual clinician on this structured vignette-based task. This places the best-performing models in a clinically meaningful intermediate performance zone.^23^

At the same time, this comparison should be interpreted cautiously. Similarity to individual clinician-level agreement in a simulated benchmark does not imply readiness for autonomous use. Human judgement in real practice is supported by contextual clarification, responsibility for resolving ambiguity, and the ability to reconsider discordant findings, whereas model outputs remained vulnerable to downstream inconsistencies in numeric computation, exception handling, and final category assignment. Moreover, an important qualitative difference remained: clinicians did not produce major two-class misclassifications, whereas several LLMs did. Taken together, these findings suggest that some LLMs may already operate within the range of individual human performance for selected components of cardiovascular risk stratification, but that their most appropriate role at present is as supervised augmentation tools rather than autonomous decision-makers.^16,17,23,30^

Reassuringly, performance was broadly consistent across Portuguese and English prompts, with no statistically significant language-related differences in the main outcomes. This is notable because less represented languages are often considered more vulnerable to performance degradation in LLMs.^31,32^ In the present study, the main challenges in cardiovascular risk stratification appeared to relate more to downstream reasoning and rule application than to language itself, although this observation should be confirmed in larger multilingual evaluations.^33,34^

## Limitations

Our study used simulated clinical vignettes rather than real clinical notes, which may limit external validity and underrepresent documentation artefacts such as abbreviations, contradictions, and missing-data patterns encountered in electronic health records. The vignette case mix was skewed towards high- and very high-risk scenarios, which may have inflated agreement metrics and may reduce generalizability to lower-prevalence settings. The sample size of 30 vignettes, although adequate for initial benchmarking, provided limited statistical power for subgroup analyses, and the resulting wide confidence intervals constrain the precision with which models can be comparatively ranked. The adjudicated reference standard, although expert-based, also introduces some subjectivity in defining the ground truth for risk classification. Furthermore, the reference standard represented an idealized benchmark derived from experts explicitly focused on rigorous calculation. This likely exceeds the more implicit and often heuristic-based risk assessment used in routine practice, meaning that the models were evaluated against a stricter threshold than is commonly encountered in real-world settings. Evaluation within a single guideline framework (ESC/SCORE2) further limits generalizability to other calculators and prevention guidelines, such as ASCVD or QRISK3. The cross-sectional, single-time-point design may not reflect the rapidly evolving nature of contemporary models. Each model was run once per language; although English and Portuguese outputs were broadly consistent, robustness to repeated sampling, temperature settings, random seeds, and prompt variation remains untested. Finally, all analyses used zero-shot prompting; few-shot prompting, chain-of-thought reasoning, fine-tuning, or retrieval augmentation might yield different results. Future work should therefore evaluate these systems in real-world electronic health record environments, examine repeated-run stability, and test whether integration with deterministic calculators, rule-based engines, or retrieval-supported workflows can improve downstream consistency and safety.^35,36^

## Conclusions

In this benchmark of eleven contemporary LLMs, models showed near-perfect extraction of traditional cardiovascular risk factors from unstructured clinical notes, but only moderate performance in ESC-aligned final risk categorization and variable numeric SCORE2 estimation. Post hoc deterministic recalculation substantially improved agreement across all models, indicating that current limitations arise mainly downstream of extraction, during formal risk computation and category assignment. LLMs may therefore already have practical value as supervised tools for information structuring and eligibility assessment, but not as autonomous end-to-end risk stratification systems. Further evaluation in real-world clinical workflows is needed before broader implementation.

## Supplementary Material

ztag073_Supplementary_Data

## Data Availability

The complete set of synthetic clinical vignettes, adjudicated labels, and model outputs that support the findings of this study are available on the Open Science Framework (OSF) at https://doi.org/10.17605/OSF.IO/J2ZK9.

## Lead author biography

José Ferreira Santos is a consultant cardiologist and Chief Medical Officer at Hospital da Luz Setúbal in Portugal. With over 20 years of experience in clinical cardiology and healthcare leadership, Dr. Santos specializes in cardiovascular imaging and preventive cardiology. He has authored 29 peer-reviewed papers and presented over 200 abstracts at international conferences and is currently a PhD student. He has a growing interest in innovative digital solutions to move forward cardiovascular prevention and is the founder of Cardio da Vida, a platform focused on cardiovascular disease prevention and public health education.

## Code availability

All R code to reproduce data processing, model evaluation, and figures is available at the same OSF project (https://doi.org/10.17605/OSF.IO/J2ZK9). Versioned releases will be archived at this DOI.
