# Supplementary material for: Large language models approach clinician performance in ESC cardiovascular risk stratification: a vignette-based benchmark study
Source: Eur Heart J Digit Health. 2026 May 20;7(5):ztag073. doi: 10.1093/ehjdh/ztag073 (PMC13215473; doi:10.1093/ehjdh/ztag073)
Supplement: ztag073_Supplementary_Data [file ztag073_supplementary_data.docx]

**Supplementary Material**

Benchmarking large language models for cardiovascular risk stratification using clinical vignettes

Index

[Supplementary Figures (Section I) 3](#_Toc225176878)

[Supplementary Figure S1. Gold-standard ESC 3-class risk categories by age and sex 3](#_Toc225176879)

[Supplementary Figure S2. Expected (a priori) vs observed (gold-standard) ESC 3-class risk distribution 4](#_Toc225176880)

[Supplementary Figure S3. Micro-averaged performance metrics across models for cardiovascular risk factors 5](#_Toc225176881)

[Supplementary Figure S4. Micro-averaged versus macro-averaged F1 Scores for cardiovascular risk factors 6](#_Toc225176882)

[Supplementary Figure S5. Confusion matrices for three-class cardiovascular risk classification across LLMs (PT+EN; κw order) 7](#_Toc225176883)

[Supplementary Figure S6. Numeric agreement with SCORE2: Bland–Altman plots by model 8](#_Toc225176884)

[Supplementary Figure S7. Bilingual consistency across key analytical domains (Portuguese vs English) 9](#_Toc225176885)

[Supplementary Tables (Section II) 10](#_Toc225176886)

[Supplementary Table S1. Specifications and configurations of LLM evaluated 10](#_Toc225176887)

[Supplementary Table S2. Evolution of prompt versions used for pilot development of the SCORE2 risk-classification workflow 11](#_Toc225176888)

[Supplementary Table S3. Performance of pilot prompt versions for SCORE2 exception detection and risk estimation 12](#_Toc225176889)

[Supplementary Table S4. Traditional cardiovascular risk factors and risk modifiers evaluated in the study 13](#_Toc225176890)

[Supplementary Table S5. Per-model extraction metrics for cardiovascular risk factors 14](#_Toc225176891)

[Supplementary Table S6. Per-model extraction metrics for SCORE2 input risk factors 15](#_Toc225176892)

[Supplementary Table S7. Pooled per-factor extraction metrics across models (cardiovascular risk factors) 16](#_Toc225176893)

[Supplementary Table S8. Per-model extraction metrics for risk modifiers 17](#_Toc225176894)

[Supplementary Table S9. Pooled per-modifier extraction metrics across models (risk modifiers) 18](#_Toc225176895)

[Supplementary Table S10. High-risk (High + Very-High) vs Low-to-Moderate — binary performance by model (ordered by κw) 19](#_Toc225176896)

[Supplementary Table S11. SCORE2 exception recognition: decision patterns, reasoning quality, and condition identification accuracy 20](#_Toc225176897)

[Supplementary Table S12. Numeric agreement with SCORE2 21](#_Toc225176898)

[Supplementary Appendices (Section III) 22](#_Toc225176899)

[Supplementary Appendix S1. Standardized Prompt Template 22](#_Toc225176900)

[Supplementary Appendix S2. Example simulated outpatient vignette 25](#_Toc225176901)

# **Supplementary Figures (Section I)**

## **Supplementary Figure S1. Gold-standard ESC 3-class risk categories by age and sex**


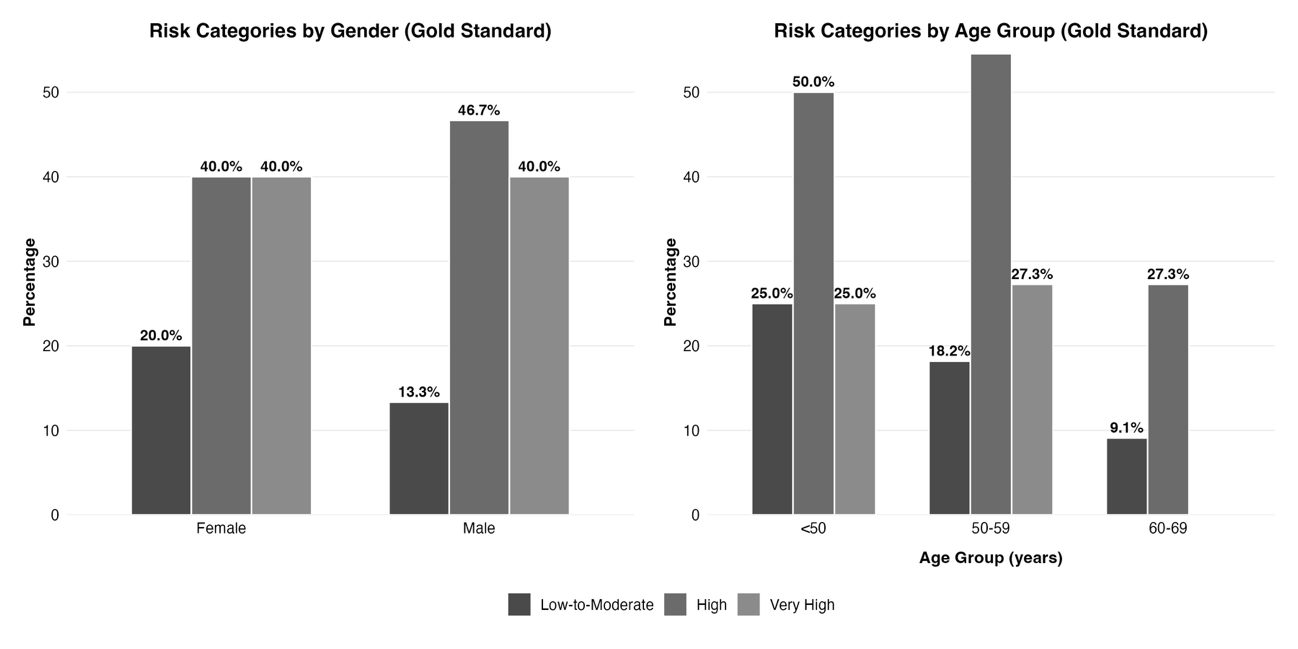


**Legend.** Distribution of gold-standard 10-year ESC cardiovascular risk categories (low-to-moderate, high, very-high) across the 30 simulated outpatient vignettes, stratified by sex (left panel) and age (right panel). Bars show absolute counts and percentages within each stratum.

## **Supplementary Figure S2. Expected (a priori) vs observed (gold-standard) ESC 3-class risk distribution**


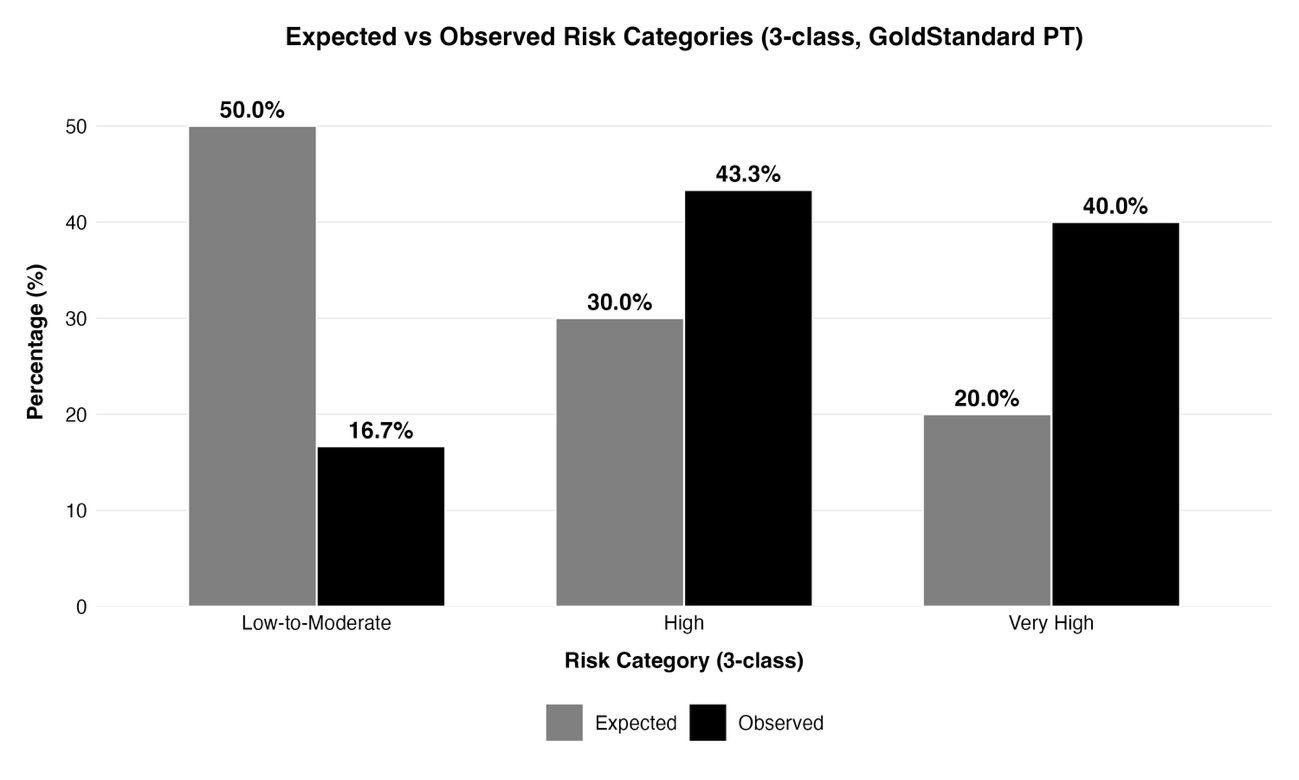


**Legend.** Comparison between the prespecified design targets for the three ESC risk categories (50% low-to-moderate, 30% high, 20% very-high) and the adjudicated gold-standard distribution across all 30 vignettes. Bars/points show proportions, with exact counts annotated for each risk category.

## **Supplementary Figure S3. Micro-averaged performance metrics across models for cardiovascular risk factors**

**
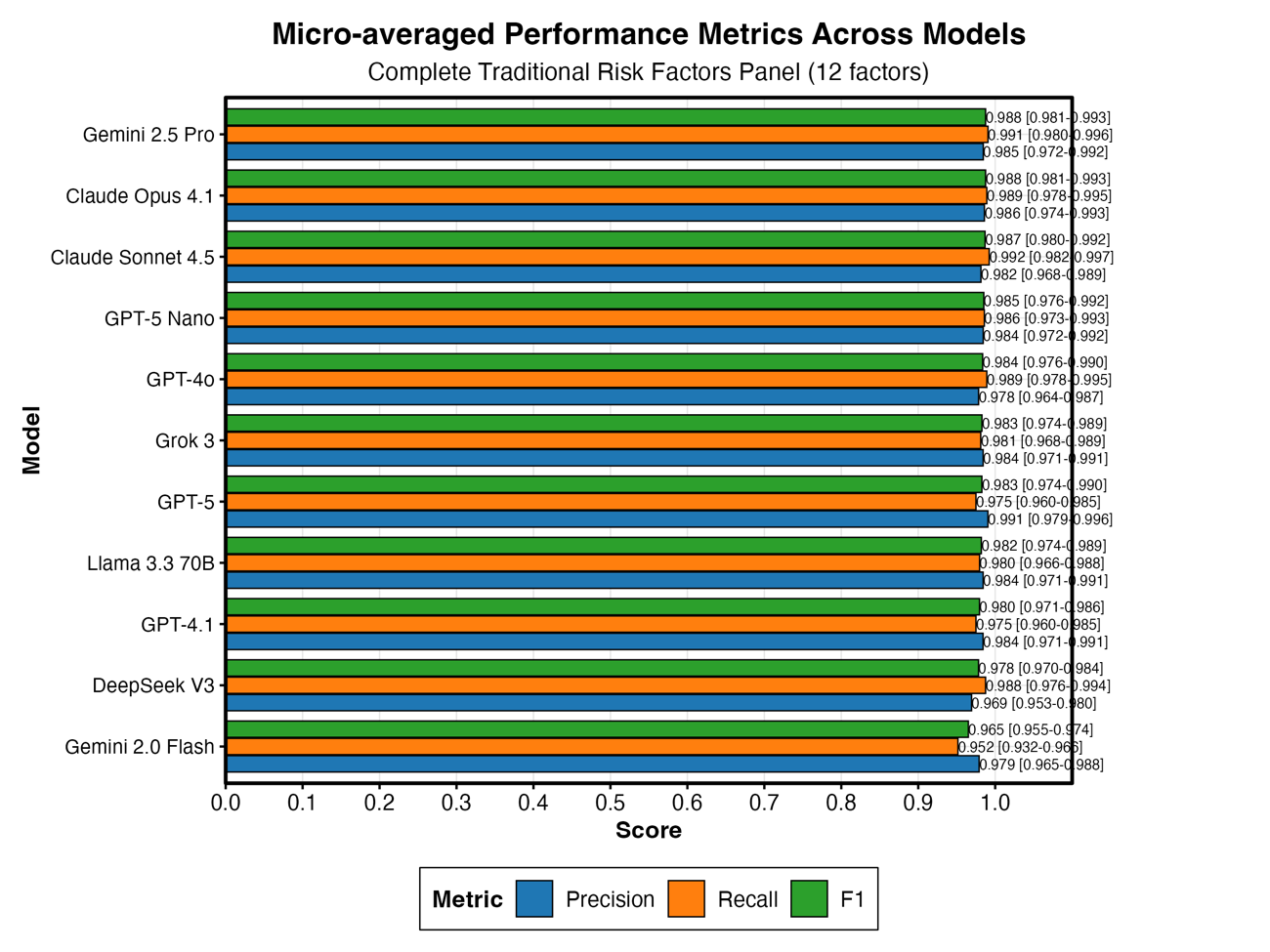
**

**Legend.** Micro-averaged extraction performance for traditional cardiovascular risk factors across all 11 large language models, pooling 60 vignettes (30 Portuguese and 30 English). The figure displays, for each model, micro-F1, micro-precision and micro-recall (with 95% confidence intervals), summarizing the results reported numerically in Table S5.

## **Supplementary Figure S4. Micro-averaged versus macro-averaged F1 Scores for cardiovascular risk factors**


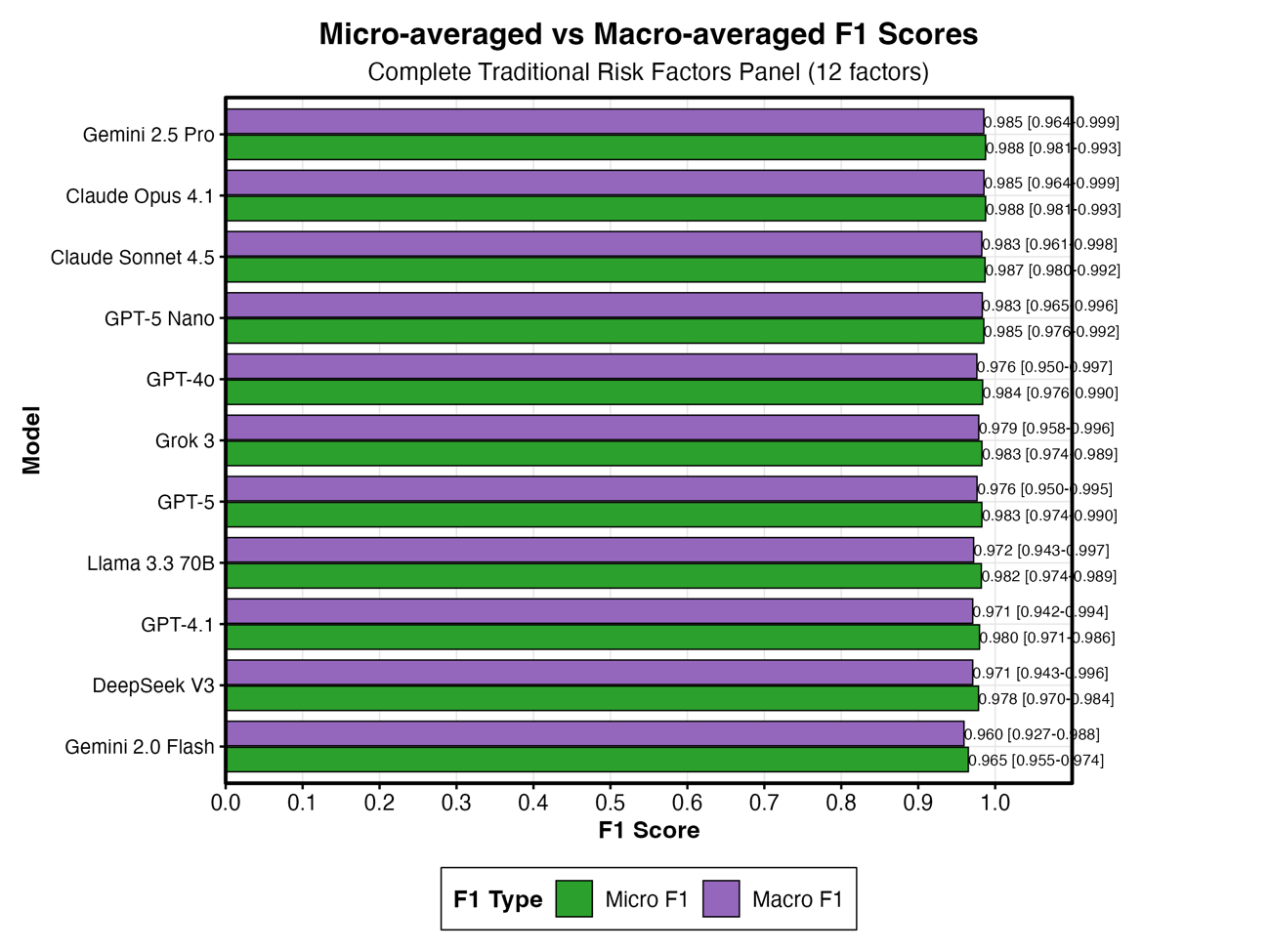


**Legend.** Micro and macro-averaged extraction performance for traditional cardiovascular risk factors across all 11 large language models, pooling 60 vignettes (30 Portuguese and 30 English). The figure displays, for each model, micro-F1 and macro F1 (with 95% confidence intervals), summarizing the results reported numerically in Table S5.

## **Supplementary Figure S5. Confusion matrices for three-class cardiovascular risk classification across LLMs (PT+EN; κw order)**


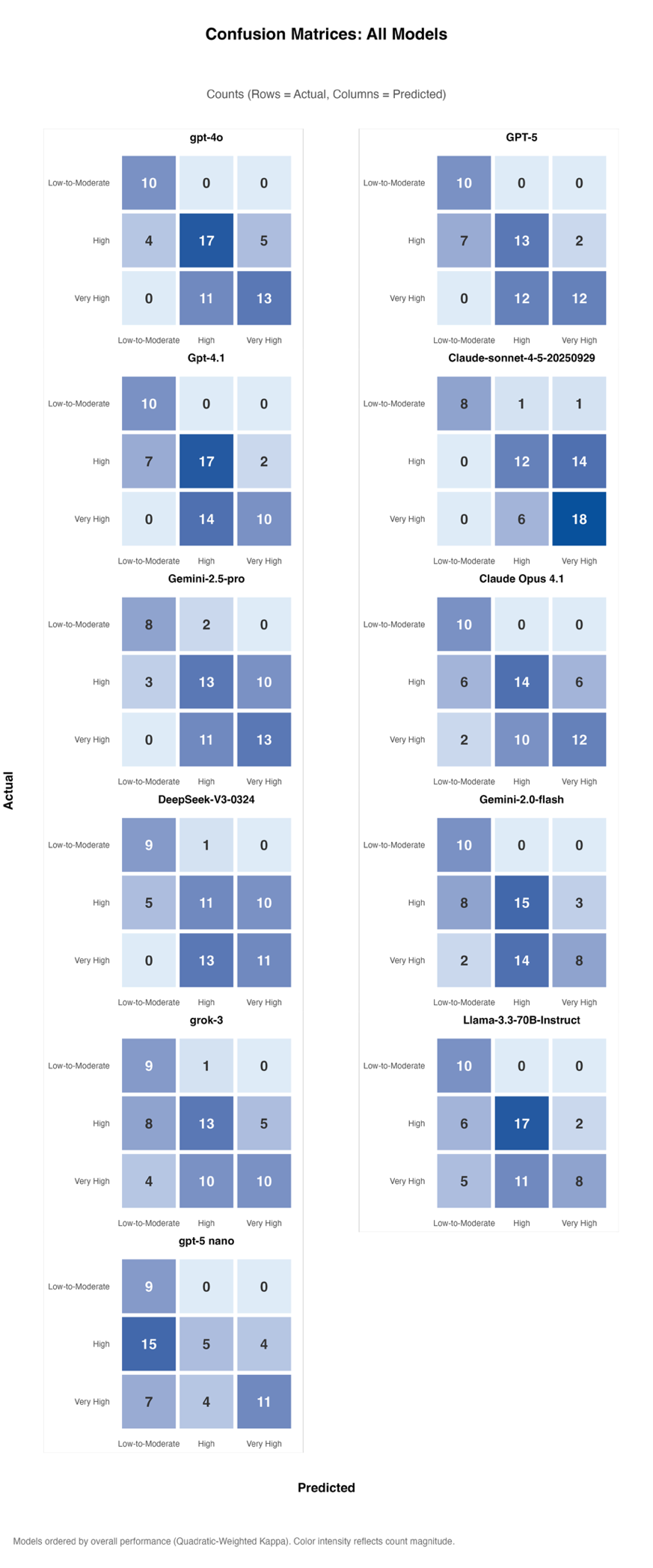


## **Supplementary Figure S6. Numeric agreement with SCORE2: Bland–Altman plots by model**


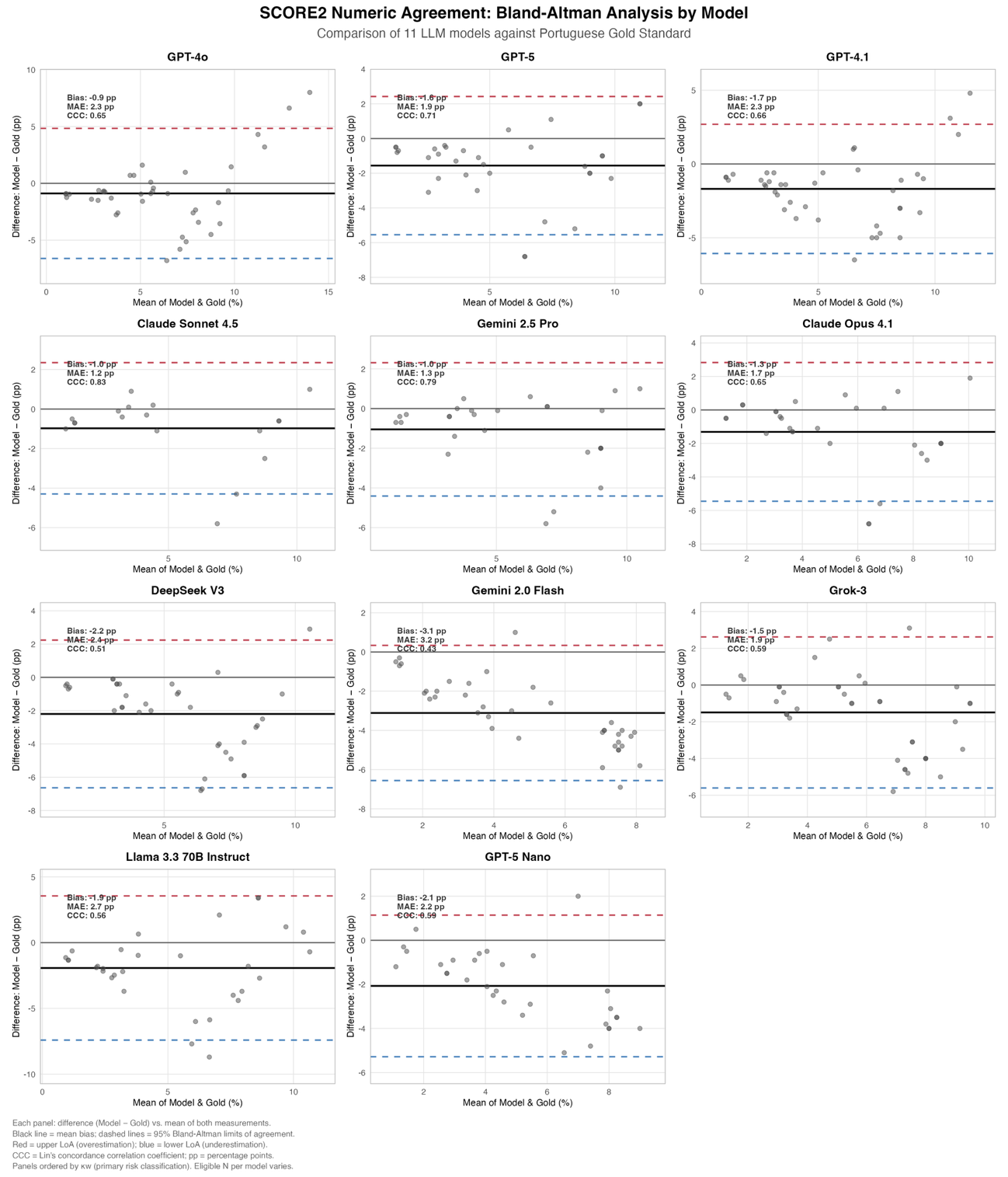


**Legend.** Bland–Altman plots for LLM-predicted vs. Gold Standard SCORE2 (Portuguese and English datasets pooled). Y-axis: difference (Model − Gold, percentage points, pp); X-axis: mean of paired estimates (%). Solid black line = mean bias; red/blue dashed lines = 95% limits of agreement (bias ±1.96 SD). Panel insets report bias, MAE (mean absolute error), and CCC (concordance correlation coefficient. Models are ordered by the primary ordinal metric (κw). N varies by model (eligible vignettes only)

## **Supplementary Figure S7. Bilingual consistency across key analytical domains (Portuguese vs English)**

**
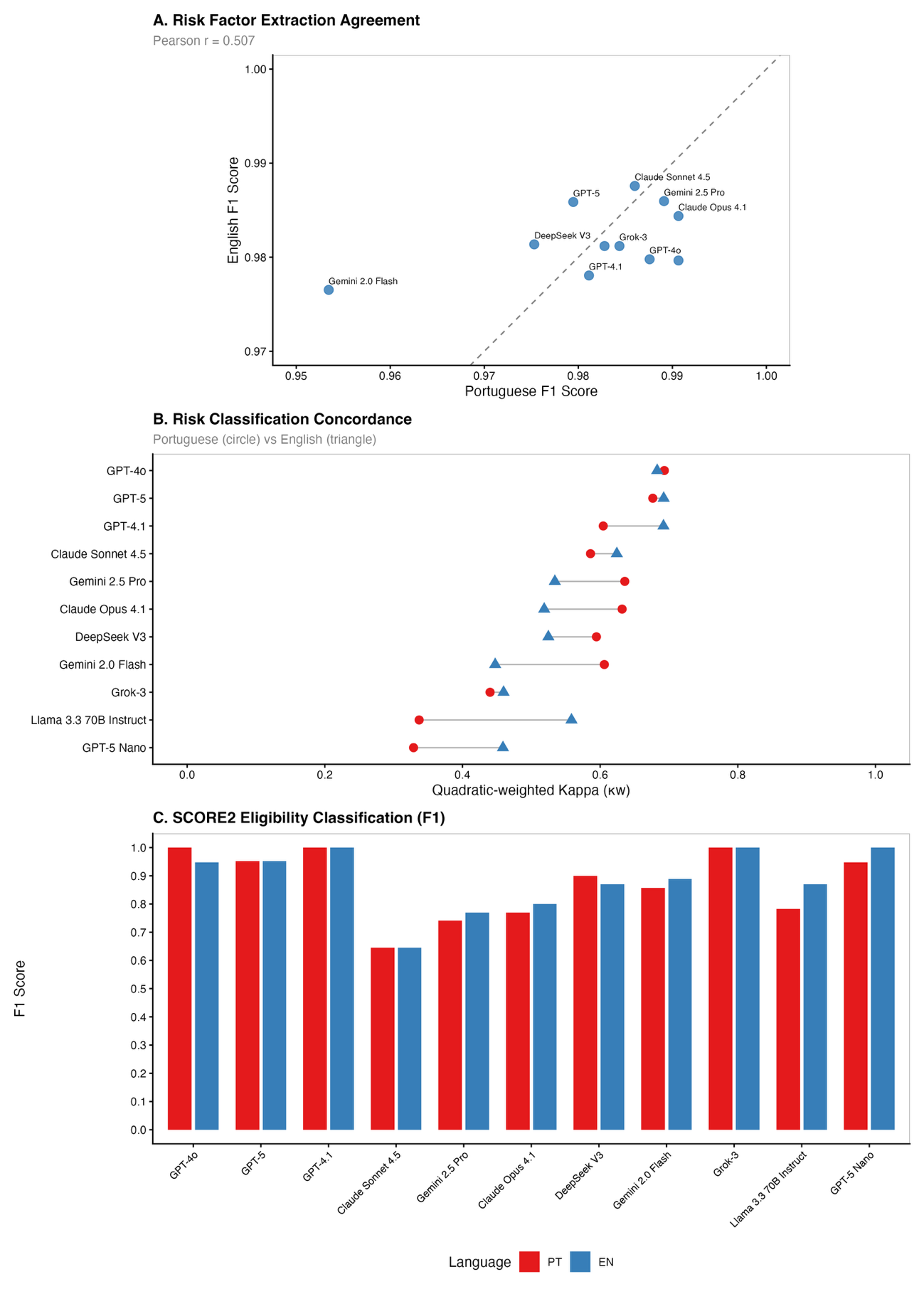
**

**Legend:** Comparison of Portuguese (PT) and English (EN) performance for 11 large language models across three prespecified domains. (A) Scatterplot of Micro-F1 for traditional risk-factor extraction (EN on y-axis, PT on x-axis); the dashed line indicates equality between languages. (B) Quadratic-weighted Cohen’s κ (κw) for ESC three-class risk classification; circles represent PT and triangles represent EN. (C) F1 for SCORE2 applicability classification, defined as the binary decision to apply SCORE2 or assign risk by exception-based classification.

# **Supplementary Tables (Section II)**

## **Supplementary Table S1.** **Specifications and configurations of LLM evaluated**

| **Model** | **Version** | **Run Date** | **Decoding Parameters** | **Inference Mode** | **Hardware / API Endpoint** |
| --- | --- | --- | --- | --- | --- |
| Claude Opus 4.1 | claude-opus-4-1-20250805 | 2025-10-04 | max_tokens = 8192; temperature = Default (≈ 0.2); top_p = Default (1.0); no stop sequences | Deterministic single-pass | Anthropic API (Python SDK) |
| Claude Sonnet 4.5 | claude-sonnet-4-5-20250929 | 2025-10-04 | max_tokens = 8192; temperature = Default (≈ 0.2); top_p = Default (1.0); no stop sequences | Deterministic single-pass | Anthropic API (Python SDK) |
| DeepSeek-V3-0324 | DeepSeek-V3-0324 (version:1) | 2025-09-24 | max_tokens = 4096; temperature = default; top_p = default | Stochastic; single-pass | Azure AI Foundry (Azure OpenAI Python SDK) |
| Gemini 2.0 Flash | gemini-2.0-flash | 2025-09-25 | Temperature: 1.0; topP: 0,95; topK: 64 (default) | Stochastic; single pass | Gemini API (Python SDK) |
| Gemini 2.5 Pro | gemini-2.5-pro | 2025-09-25 | Temperature:1.0; top:0.95; topK:64 (default) | Stochastic; single pass | Gemini API (Python SDK) |
| GPT-4.1 | gpt-4.1 (version:2025-04-14) | 2025-08-26 | max_tokens = 4096; temperature = default; top_p = default | Stochastic; single-pass | Azure AI Foundry (Azure OpenAI Python SDK) |
| GPT-4o | gpt-4o (version:2024-11-20) | 2025-08-27 | max_tokens = 4096; temperature = default; top_p = default) | Stochastic; single-pass | Azure AI Foundry (Azure OpenAI Python SDK) |
| GPT-5 | gpt-5^a^ | 2025-09-25 | Max_tokens=none; temperature =1.0: top_p=1.0 (default) | Stochastic; single pass | OpenAI API (Python SDK) |
| GPT-5 nano | gpt-5-nano | 2025-09-25 | Max_tokens=none; temperature =1.0: top_p=1.0 (default) | Stochastic; single pass | OpenAI API (Python SDK) |
| Grok-3 | grok-3 (version:1) | 2025-08-27 | max_tokens = 4096; temperature = default; top_p = default | Stochastic; single-pass | Azure AI Foundry (Azure OpenAI Python SDK) |
| Llama-3.3-70B-Instruct | Llama-3.3-70B-Instruct (version:5) | 2025-09-23 | max_tokens = 4096; temperature = default; top_p = default | Stochastic; single-pass | Azure AI Foundry (Azure OpenAI Python SDK) |

**Legend.** Specifications of the 11 large language models (LLMs) included in the study, detailing model identifier and version, run date, decoding parameters (e.g. temperature, top_p, max_tokens), inference mode (stochastic vs deterministic, single-pass), and hardware or API endpoint used for inference. ^a^ extended thinking/reasoning mode was not activated.

## **Supplementary Table S2.** **Evolution of prompt versions used for pilot development of the SCORE2 risk-classification workflow**

| **Version** | **Risk factor extraction** | **SCORE2 exceptions** | **SCORE2 calculation methods** | **Pilot-tested models** |
| --- | --- | --- | --- | --- |
| **V1.0** | Single broad extraction framework from the start. Requires a structured Markdown table with four fixed domains: Traditional Risk Factors, Lifestyle, Associated Diseases, and Risk Modifiers. Includes extensive variable list beyond SCORE2 core inputs. | Checks whether SCORE2 is applicable in patients aged 40–69 and excludes those with CVD, CKD, diabetes, familial dyslipidemia, or other exclusions. If not applicable, patient should be classified according to ESC exceptions (exception classes are described in a relatively broad way). | Flexible approach: SCORE2 may be calculated using ESC table, published original formula, or a recognized online calculator for moderate-risk countries; the model must specify which method was used. | PT |
| **V2.0** | Two-step extraction structure. First, initial minimal extraction limited to SCORE2 inputs: age, sex, smoking, systolic BP, and non-HDL cholesterol; later followed by a broader four-domain extraction.  and modifiers. | Checks whether SCORE2 is applicable (Yes/No) and asks for the exception if present (no definitions) | Same as previous | PT + EN |
| **V3.0** | Same as previous | SCORE2 exceptions explicitly operationalized: Diabetes, Familial Hypercholesterolemia/Familial Dyslipidemia, CKD, and Atherosclerotic Cardiovascular Disease are listed as the possible override categories. | SCORE2 should be calculated exclusively with the official ESC online calculator for moderate-risk countries, and only using the factors extracted in step 1 | PT + EN |
| **V4.0 (Final)** | Still two-step extraction. The later broader extraction is simplified to two domains: Risk Factors and Risk Modifiers. | Same as previous | SCORE2 should be calculated using only extracted variables, with either the official ESC online calculator or, alternatively, the moderate-risk SCORE2 table in the ESC guidelines. Subjective approximation is explicitly forbidden. | PT + EN |

**Legend:** Table summarizing the main structural differences across sequential prompt versions developed for pilot testing of LLM-based cardiovascular risk assessment. Versions are compared according to risk-factor extraction strategy, handling of SCORE2 exceptions, SCORE2 calculation instructions, and language of pilot testing. Only prompt versions incorporating substantive changes to analytical logic were retained and reported; intermediate drafts with minor editorial refinements were not evaluated separately. Full prompts versions available at Open Science Framework (https://doi.org/10.17605/OSF.IO/J2ZK9)

## **Supplementary Table S3.** **Performance of pilot prompt versions for SCORE2 exception detection and risk estimation**

| **Prompt Version** | **Model** | **n** | **SCORE2 Exceptions (%)** | **MAE**  **(95% CI)** | **Bias**  **(95% CI)** | **κw**  **(95% CI)** |
| --- | --- | --- | --- | --- | --- | --- |
| V1.0 ᵃ | Gemini-2.0-Flash | 30 | N/A | 3.00  [1.72–4.33] | -2.89  [-4.32 to -1.48] | 0.440  [-0.031–0.718] |
| V2.0 | GPT-4o | 60 | 75% | 2.03  [1.39–2.72] | -1.52  [-2.35 to -0.74] | 0.328  [0.157–0.483] |
| V2.0 | Gemini-2.0-Flash | 60 | 83.3% | 3.14  [2.36–3.94] | -3.08  [-3.90 to -2.29] | 0.448  [0.166–0.644] |
| V3.0 | Gemini-2.0-Flash | 60 | 90% | 3.43  [2.65–4.22] | -3.43  [-4.22 to -2.65] | 0.438  [0.232–0.605] |
| V4.0 (final) | GPT-4o | 60 | 98.3% | 2.27  [1.53–3.04] | -0.89  [-1.89 to 0.11] | 0.688  [0.442–0.842] |
| V4.0 (final) | Gemini-2.0-Flash | 60 | 91.7% | 3.16  [2.44–3.87] | -3.11  [-3.84 to -2.34] | 0.523  [0.288–0.703] |

**Legend:** Pilot testing was limited to prompt versions incorporating substantive changes to risk-factor extraction, SCORE2 exception handling, or SCORE2 calculation instructions; intermediate drafts with only minor editorial refinements were not evaluated separately. Data are shown by prompt version and model. SCORE2 Exceptions (%) indicates accuracy for identifying exclusion from direct SCORE2 calculation; MAE and Bias represent agreement with the reference numeric SCORE2 value in SCORE2-applicable cases only, with negative Bias indicating underestimation; κw denotes quadratic-weighted Cohen’s kappa for agreement in final ESC risk category. Ninety-five percent confidence intervals were obtained by vignette-level paired bootstrap (2000 iterations). ᵃV1.0 was tested only in Portuguese and did not require structured reporting of SCORE2 exception status; therefore, exception accuracy was not available.

## **Supplementary Table S4.** **Traditional cardiovascular risk factors and risk modifiers evaluated in the study**

| **Domain** | **Variable** |
| --- | --- |
| Traditional cardiovascular risk factors  (SCORE2 input variable) | Age |
|  | Sex |
|  | Smoking status |
|  | Systolic blood pressure |
|  | Total cholesterol |
|  | HDL cholesterol |
| Other Traditional cardiovascular risk factors | Diastolic blood pressure |
|  | LDL cholesterol |
|  | Non-HDL cholesterol |
|  | Triglycerides |
|  | Hypertension diagnosis |
|  | Dyslipidaemia diagnosis |
| Risk modifiers | Elevated coronary calcium score |
|  | Calcium score = 0 |
|  | Pre-diabetes |
|  | Obesity |
|  | Family history of premature ASCVD |
|  | Elevated lipoprotein(a) |
|  | Increased arterial stiffness |
|  | Elevated high-sensitivity C-reactive protein |
|  | Chronic inflammatory disease |
|  | Obstructive sleep apnoea |
|  | Chronic obstructive pulmonary disease |
|  | Cancer |

## **Supplementary Table S5. Per-model extraction metrics for cardiovascular risk factors**

| **Model** | **Micro-F1 (95% CI)** | **Micro-Precision (95% CI)** | **Micro-Recall (95% CI)** | **Macro-F1 (95% CI)** | **Macro-Precision (95% CI)** | **Macro-Recall (95% CI)** | **Jaccard (95% CI)** |
| --- | --- | --- | --- | --- | --- | --- | --- |
| **GPT-4o** | 0.98 [0.98, 0.99] | 0.98 [0.96, 0.99] | 0.99 [0.98, 0.99] | 0.98 [0.95, 1.00] | 0.97 [0.92, 1.00] | 0.98 [0.97, 1.00] | 0.97 [0.95, 0.98] |
| **GPT-5** | 0.98 [0.97, 0.99] | 0.99 [0.98, 1.00] | 0.97 [0.96, 0.98] | 0.98 [0.95, 0.99] | 0.99 [0.96, 1.00] | 0.97 [0.94, 0.99] | 0.97 [0.95, 0.98] |
| **GPT-4.1** | 0.98 [0.97, 0.99] | 0.98 [0.97, 0.99] | 0.97 [0.96, 0.98] | 0.97 [0.94, 0.99] | 0.97 [0.94, 1.00] | 0.97 [0.94, 0.99] | 0.96 [0.94, 0.97] |
| **Claude Sonnet 4.5** | 0.99 [0.98, 0.99] | 0.98 [0.97, 0.99] | 0.99 [0.98, 1.00] | 0.98 [0.96, 1.00] | 0.98 [0.94, 1.00] | 0.99 [0.98, 1.00] | 0.97 [0.96, 0.98] |
| **Gemini 2.5 Pro** | 0.99 [0.98, 0.99] | 0.98 [0.97, 0.99] | 0.99 [0.98, 1.00] | 0.99 [0.96, 1.00] | 0.98 [0.95, 1.00] | 0.99 [0.98, 1.00] | 0.98 [0.96, 0.99] |
| **Claude Opus 4.1** | 0.99 [0.98, 0.99] | 0.99 [0.97, 0.99] | 0.99 [0.98, 0.99] | 0.99 [0.96, 1.00] | 0.98 [0.96, 1.00] | 0.99 [0.97, 1.00] | 0.98 [0.96, 0.99] |
| **DeepSeek V3** | 0.98 [0.97, 0.98] | 0.97 [0.95, 0.98] | 0.99 [0.98, 0.99] | 0.97 [0.94, 1.00] | 0.96 [0.92, 0.99] | 0.99 [0.97, 1.00] | 0.96 [0.94, 0.97] |
| **Gemini 2.0 Flash** | 0.97 [0.95, 0.97] | 0.98 [0.96, 0.99] | 0.95 [0.93, 0.97] | 0.96 [0.93, 0.99] | 0.97 [0.93, 1.00] | 0.95 [0.90, 0.99] | 0.93 [0.92, 0.95] |
| **Grok 3** | 0.98 [0.97, 0.99] | 0.98 [0.97, 0.99] | 0.98 [0.97, 0.99] | 0.98 [0.96, 1.00] | 0.98 [0.95, 1.00] | 0.98 [0.96, 0.99] | 0.97 [0.95, 0.98] |
| **Llama 3.3 70B** | 0.98 [0.97, 0.99] | 0.98 [0.97, 0.99] | 0.98 [0.97, 0.99] | 0.97 [0.94, 1.00] | 0.98 [0.95, 1.00] | 0.97 [0.92, 0.99] | 0.96 [0.95, 0.98] |
| **GPT-5 Nano** | 0.99 [0.98, 0.99] | 0.98 [0.97, 0.99] | 0.99 [0.97, 0.99] | 0.98 [0.96, 1.00] | 0.98 [0.95, 1.00] | 0.99 [0.97, 1.00] | 0.97 [0.96, 0.98] |

**Legend.** Per-model performance for extraction of all predefined cardiovascular risk factors across 60 vignettes (30 Portuguese and 30 English). Metrics include Micro-F1, Micro-precision, Micro-recall, Macro-F1, Macro-precision, Macro-recall, and mean Jaccard index, each reported with 95% confidence intervals.

## **Supplementary Table S6. Per-model extraction metrics for SCORE2 input risk factors**

| **Model** | **Micro-F1**  **(CI 95%)** | **Micro-Precision (CI 95%)** | **Micro-Recall**  **(CI 95%)** | **Macro-F1 (CI 95%)** | **Macro-Precision (CI 95%)** | **Macro-Recall**  **(CI 95%)** | **Jaccard**  **(CI 95%)** |
| --- | --- | --- | --- | --- | --- | --- | --- |
| **GPT-4o** | 0.99 [0.98, 1.00] | 0.99 [0.97, 1.00] | 0.99 [0.98, 1.00] | 0.99 [0.98, 1.00] | 0.99 [0.97, 1.00] | 0.99 [0.98, 1.00] | 0.98 [0.97, 0.99] |
| **GPT-5** | 0.98 [0.97, 0.99] | 1.00 [0.99, 1.00] | 0.97 [0.95, 0.98] | 0.98 [0.96, 1.00] | 1.00 [1.00, 1.00] | 0.97 [0.92, 1.00] | 0.97 [0.95, 0.98] |
| **GPT-4.1** | 0.99 [0.98, 0.99] | 0.99 [0.97, 1.00] | 0.98 [0.96, 0.99] | 0.99 [0.97, 1.00] | 0.99 [0.97, 1.00] | 0.98 [0.96, 1.00] | 0.98 [0.96, 0.99] |
| **Claude Sonnet 4.5** | 0.99 [0.99, 1.00] | 0.99 [0.98, 1.00] | 0.99 [0.98, 1.00] | 0.99 [0.98, 1.00] | 0.99 [0.98, 1.00] | 0.99 [0.98, 1.00] | 0.99 [0.97, 1.00] |
| **Gemini 2.5 Pro** | 0.99 [0.98, 0.99] | 1.00 [0.98, 1.00] | 0.98 [0.96, 0.99] | 0.99 [0.97, 1.00] | 1.00 [0.99, 1.00] | 0.98 [0.95, 1.00] | 0.98 [0.96, 0.99] |
| **Claude Opus 4.1** | 0.99 [0.98, 0.99] | 1.00 [0.99, 1.00] | 0.98 [0.95, 0.99] | 0.99 [0.97, 1.00] | 1.00 [1.00, 1.00] | 0.97 [0.94, 1.00] | 0.98 [0.96, 0.99] |
| **DeepSeek V3** | 0.99 [0.98, 1.00] | 0.99 [0.97, 1.00] | 0.99 [0.98, 1.00] | 0.99 [0.98, 1.00] | 0.99 [0.97, 1.00] | 0.99 [0.98, 1.00] | 0.98 [0.97, 0.99] |
| **Gemini 2.0 Flash** | 0.99 [0.98, 0.99] | 0.99 [0.97, 1.00] | 0.98 [0.96, 0.99] | 0.99 [0.97, 1.00] | 0.99 [0.97, 1.00] | 0.98 [0.95, 1.00] | 0.97 [0.96, 0.99] |
| **Grok-3** | 0.98 [0.97, 0.99] | 1.00 [0.99, 1.00] | 0.97 [0.95, 0.98] | 0.98 [0.96, 1.00] | 1.00 [1.00, 1.00] | 0.97 [0.93, 1.00] | 0.97 [0.96, 0.99] |
| **Llama 3.3 70B Instruct** | 0.98 [0.97, 0.99] | 1.00 [0.99, 1.00] | 0.97 [0.95, 0.98] | 0.98 [0.95, 1.00] | 1.00 [1.00, 1.00] | 0.97 [0.91, 1.00] | 0.98 [0.97, 0.99] |
| **GPT-5 nano** | 0.99 [0.98, 1.00] | 1.00 [0.98, 1.00] | 0.99 [0.97, 0.99] | 0.99 [0.98, 1.00] | 1.00 [0.99, 1.00] | 0.99 [0.96, 1.00] | 0.98 [0.97, 0.99] |

**Legend.** Extraction performance by model for SCORE2 input variables (age, sex, smoking status, systolic blood pressure, total cholesterol, HDL cholesterol, and non-HDL cholesterol) across 60 vignettes. Micro- and macro-averaged F1, precision, recall, and Jaccard index are presented with 95% confidence intervals for each LLM.

## **Supplementary Table S7. Pooled per-factor extraction metrics across models (cardiovascular risk factors)**

| **Factor** | **F1 (95% CI)** | **Precision (95% CI)** | **Recall (95% CI)** | **N determinable** | **N excluded** |
| --- | --- | --- | --- | --- | --- |
| **Age** | 1.00 [1.00, 1.00] | 1.00 [0.99, 1.00] | 1.00 [0.99, 1.00] | 660 | 0 |
| **Sex** | 1.00 [1.00, 1.00] | 1.00 [0.99, 1.00] | 1.00 [0.99, 1.00] | 660 | 0 |
| **Systolic BP** | 0.97 [0.96, 0.98] | 1.00 [0.99, 1.00] | 0.95 [0.93, 0.96] | 660 | 0 |
| **Diastolic BP** | 0.99 [0.98, 0.99] | 1.00 [0.99, 1.00] | 0.98 [0.96, 0.98] | 660 | 0 |
| **Total Cholesterol** | 1.00 [1.00, 1.00] | 1.00 [0.99, 1.00] | 1.00 [0.99, 1.00] | 660 | 0 |
| **HDL Cholesterol** | 1.00 [1.00, 1.00] | 1.00 [0.99, 1.00] | 1.00 [0.99, 1.00] | 660 | 0 |
| **Non-HDL-Chol** | 0.98 [0.98, 0.99] | 1.00 [0.99, 1.00] | 0.97 [0.95, 0.98] | 660 | 0 |
| **LDL Cholesterol** | 1.00 [1.00, 1.00] | 1.00 [0.99, 1.00] | 1.00 [0.99, 1.00] | 660 | 0 |
| **Triglycerides** | 1.00 [1.00, 1.00] | 1.00 [0.99, 1.00] | 1.00 [0.99, 1.00] | 660 | 0 |
| **Hypertension** | 0.96 [0.94, 0.97] | 0.97 [0.95, 0.98] | 0.94 [0.92, 0.96] | 660 | 0 |
| **Dyslipidemia** | 0.88 [0.86, 0.90] | 0.82 [0.79, 0.85] | 0.94 [0.92, 0.96] | 655 | 5 |
| **Smoking** | 0.94 [0.91, 0.96] | 0.92 [0.88, 0.95] | 0.95 [0.92, 0.97] | 636 | 24 |

**Legend.** Factor-level extraction performance aggregated across all 11 LLMs for each traditional cardiovascular risk factor. For every factor, the table reports F1, precision, and recall with 95% confidence intervals, alongside the number of vignettes in which the factor was determinable and the number excluded.

## **Supplementary Table S8. Per-model extraction metrics for risk modifiers**

| **Model** | **Micro-F1 (CI 95%)** | **Micro-Precision (CI 95%)** | **Micro-Recall (CI 95%)** | **Macro-F1 (CI 95%)** | **Macro-Precision (CI 95%)** | **Macro-Recall (CI 95%)** | **Jaccard (CI 95%)** |
| --- | --- | --- | --- | --- | --- | --- | --- |
| **GPT-4o** | 0.77 [0.66, 0.87] | 0.73 [0.57, 0.87] | 0.83 [0.70, 0.95] | 0.68 [0.40, 0.69] | 0.70 [0.38, 0.74] | 0.75 [0.42, 0.74] | 0.76 [0.66, 0.86] |
| **GPT-5** | 0.80 [0.67, 0.90] | 0.80 [0.65, 0.92] | 0.81 [0.66, 0.94] | 0.78 [0.37, 0.81] | 0.81 [0.39, 0.83] | 0.79 [0.37, 0.82] | 0.76 [0.63, 0.87] |
| **GPT-4.1** | 0.74 [0.61, 0.84] | 0.74 [0.59, 0.88] | 0.74 [0.60, 0.88] | 0.65 [0.36, 0.66] | 0.71 [0.37, 0.72] | 0.67 [0.36, 0.69] | 0.70 [0.56, 0.82] |
| **Claude Sonnet 4.5** | 0.58 [0.45, 0.68] | 0.52 [0.36, 0.66] | 0.66 [0.56, 0.75] | 0.70 [0.35, 0.71] | 0.75 [0.36, 0.79] | 0.79 [0.40, 0.78] | 0.47 [0.35, 0.59] |
| **Gemini 2.5 Pro** | 0.81 [0.70, 0.90] | 0.68 [0.54, 0.81] | 1.00 [1.00, 1.00] | 0.80 [0.45, 0.83] | 0.72 [0.40, 0.77] | 1.00 [0.50, 1.00] | 0.74 [0.61, 0.86] |
| **Claude Opus 4.1** | 0.64 [0.47, 0.77] | 0.58 [0.40, 0.73] | 0.72 [0.55, 0.86] | 0.75 [0.36, 0.77] | 0.75 [0.37, 0.79] | 0.87 [0.42, 0.86] | 0.52 [0.38, 0.67] |
| **DeepSeek V3** | 0.67 [0.52, 0.78] | 0.59 [0.43, 0.74] | 0.78 [0.62, 0.90] | 0.64 [0.33, 0.66] | 0.63 [0.32, 0.66] | 0.76 [0.38, 0.76] | 0.63 [0.50, 0.76] |
| **Gemini 2.0 Flash** | 0.67 [0.54, 0.77] | 0.55 [0.41, 0.68] | 0.86 [0.75, 0.96] | 0.68 [0.37, 0.69] | 0.63 [0.34, 0.67] | 0.84 [0.46, 0.82] | 0.55 [0.41, 0.69] |
| **Grok-3** | 0.60 [0.43, 0.73] | 0.55 [0.36, 0.71] | 0.67 [0.50, 0.83] | 0.56 [0.30, 0.58] | 0.56 [0.30, 0.61] | 0.67 [0.35, 0.69] | 0.53 [0.38, 0.68] |
| **Llama 3.3 70B Instruct** | 0.67 [0.52, 0.78] | 0.56 [0.40, 0.71] | 0.83 [0.71, 0.92] | 0.77 [0.39, 0.79] | 0.76 [0.39, 0.81] | 0.91 [0.46, 0.90] | 0.56 [0.41, 0.70] |
| **GPT-5 nano** | 0.82 [0.69, 0.90] | 0.73 [0.58, 0.86] | 0.93 [0.81, 1.00] | 0.79 [0.39, 0.82] | 0.77 [0.38, 0.82] | 0.93 [0.46, 0.92] | 0.74 [0.61, 0.86] |

**Legend.** Per-model performance for extraction of predefined cardiovascular risk modifiers. The table reports Micro-F1, Micro-precision, Micro-recall, Macro-F1, Macro-precision, Macro-recall, and Jaccard index with 95% confidence intervals, pooling results across 60 bilingual vignettes.

## **Supplementary Table S9. Pooled per-modifier extraction metrics across models (risk modifiers)**

| **Modifier** | **N positives (Gold)** | **F1 (CI 95%)** | **Precision (CI 95%)** | **Recall (CI 95%)** | **N determinable** | **N excluded** |
| --- | --- | --- | --- | --- | --- | --- |
| **Calcium Score = 0** | 22 | 0.96 [0.88, 1.00] | 0.92 [0.74, 0.98] | 1.00 [0.85, 1.00] | 660 | 0 |
| **Cancer** | 44 | 0.87 [0.78, 0.94] | 1.00 [0.90, 1.00] | 0.77 [0.63, 0.87] | 660 | 0 |
| **Inflammatory Disease** | 22 | 0.61 [0.40, 0.77] | 0.58 [0.39, 0.76] | 0.64 [0.43, 0.80] | 660 | 0 |
| **COPD** | 22 | 0.92 [0.82, 0.98] | 0.85 [0.67, 0.94] | 1.00 [0.85, 1.00] | 660 | 0 |
| **Elevated Calcium Score** | 88 | 0.88 [0.84, 0.93] | 0.80 [0.71, 0.86] | 0.99 [0.94, 1.00] | 660 | 0 |
| **Elevated hs-CRP** | 22 | 0.81 [0.67, 0.93] | 1.00 [0.80, 1.00] | 0.68 [0.47, 0.84] | 660 | 0 |
| **Elevated Lp(a)** | 22 | 0.43 [0.26, 0.60] | 0.38 [0.23, 0.56] | 0.50 [0.31, 0.69] | 660 | 0 |
| **Family History of ASCVD** | 22 | 0.32 [0.21, 0.42] | 0.19 [0.13, 0.28] | 0.96 [0.78, 0.99] | 660 | 0 |
| **Increased Arterial Stiffness** | 22 | 0.71 [0.52, 0.83] | 0.65 [0.46, 0.81] | 0.77 [0.57, 0.90] | 660 | 0 |
| **Obesity** | 44 | 0.45 [0.36, 0.53] | 0.29 [0.23, 0.37] | 1.00 [0.92, 1.00] | 660 | 0 |
| **Obstructive Sleep Apnea** | 66 | 0.87 [0.80, 0.93] | 0.98 [0.90, 1.00] | 0.79 [0.68, 0.87] | 660 | 0 |
| **Pre-Diabetes** | 242 | 0.76 [0.72, 0.81] | 0.82 [0.76, 0.86] | 0.72 [0.66, 0.77] | 660 | 0 |

**Legend.** Modifier-level extraction performance aggregated across all 11 LLMs for each predefined risk modifier. The table shows the number of positive cases in the gold standard, F1, precision, and recall (with 95% confidence intervals), plus the number of vignettes in which each modifier was determinable or excluded.

## **Supplementary Table S10. High-risk (High + Very-High) vs Low-to-Moderate — binary performance by model (ordered by κw)**

| **Model** | **N / Unknown (%)** | **High-risk prevalence (%)** | **Sensitivity**  **(95% CI)** | **Specificity**  **(95% CI)** | **PPV**  **(95% CI)** | **NPV**  **(95% CI)** |
| --- | --- | --- | --- | --- | --- | --- |
| GPT-4o | 60 / 0.0% | 83.3 | 92.0  (81.2, 96.8) | 100.0  (72.2, 100.0) | 100.0  (92.3, 100.0) | 71.4  (45.4, 88.3) |
| GPT-5 | 56 / 6.7% | 82.1 | 84.8  (71.8, 92.4) | 100.0  (72.2, 100.0) | 100.0  (91.0, 100.0) | 58.8  (36.0, 78.4) |
| GPT-4.1 | 60 / 0.0% | 83.3 | 86.0  (73.8, 93.0) | 100.0  (72.2, 100.0) | 100.0  (91.8, 100.0) | 58.8  (36.0, 78.4) |
| Claude Sonnet 4.5 | 60 / 0.0% | 83.3 | 100.0  (92.9, 100.0) | 80.0  (49.0, 94.3) | 96.2  (87.0, 98.9) | 100.0  (67.6, 100.0) |
| Gemini 2.5 Pro | 60 / 0.0% | 83.3 | 94.0  (83.8, 97.9) | 80.0  (49.0, 94.3) | 95.9  (86.3, 98.9) | 72.7  (43.4, 90.3) |
| Claude Opus 4.1 | 60 / 0.0% | 83.3 | 84.0  (71.5, 91.7) | 100.0  (72.2, 100.0) | 100.0  (91.6, 100.0) | 55.6  (33.7, 75.4) |
| DeepSeek V3 | 60 / 0.0% | 83.3 | 90.0  (78.6, 95.7) | 90.0  (59.6, 98.2) | 97.8  (88.7, 99.6) | 64.3  (38.8, 83.7) |
| Gemini 2.0 Flash | 60 / 0.0% | 83.3 | 80.0  (67.0, 88.8) | 100.0  (72.2, 100.0) | 100.0  (91.2, 100.0) | 50.0  (29.9, 70.1) |
| Grok-3 | 60 / 0.0% | 83.3 | 76.0  (62.6, 85.7) | 90.0  (59.6, 98.2) | 97.4  (86.8, 99.5) | 42.9  (24.5, 63.5) |
| Llama 3.3 70B Instruct | 59 / 1.7% | 83.1 | 77.6  (64.1, 87.0) | 100.0  (72.2, 100.0) | 100.0  (90.8, 100.0) | 47.6  (28.3, 67.6) |
| GPT-5 nano | 55 / 8.3% | 83.6 | 52.2  (38.1, 65.9) | 100.0  (70.1, 100.0) | 100.0  (86.2, 100.0) | 29.0  (16.1, 46.6) |

**Legend.** Binary endpoint: High risk (High + Very-High) vs Low-to-Moderate per Gold Standard; pooled Portuguese and English (PT+EN). Models are ordered by the primary ordinal metric (κw) from Table 3. N / Unknown (%) = number of evaluated predictions / share of missing predictions out of 60 vignettes (30 PT + 30 EN). High-risk prevalence (%) is the Gold Standard proportion of High + Very-High within the evaluated set for each model (may differ slightly across models due to N differences). Sensitivity, specificity, PPV, and NPV are point estimates with 95% CIs (percentages shown to one decimal) and are calculated only on evaluated cases (unknown/abstain excluded from their denominators).

## **Supplementary Table S11.** **SCORE2 exception recognition: decision patterns, reasoning quality, and condition identification accuracy**

| Model | SCORE2 withheld (n/N; %) | Reason Provided (%) | Valid Reason (%) | Micro-F1  (95% CI) | Macro-F1  (95% CI) |
| --- | --- | --- | --- | --- | --- |
| GPT-4o | 19/60; 31.7 | 95.0 | 100.0 | 0.97 (0.92–1.00) | 0.98 (0.48–1.00) |
| GPT-5 | 22/60; 36.7 | 100.0 | 100.0 | 0.95 (0.83–1.00) | 0.97 (0.47–1.00) |
| GPT-4.1 | 20/60; 33.3 | 100.0 | 100.0 | 1.00 (1.00–1.00) | 1.00 (0.50–1.00) |
| Claude Sonnet 4.5 | 42/60; 70.0 | 100.0 | 85.7 | 0.65 (0.42–0.81) | 0.80 (0.33–0.90) |
| Gemini 2.5 Pro | 33/60; 55.0 | 100.0 | 87.9 | 0.76 (0.53–0.90) | 0.86 (0.41–0.95) |
| Claude Opus 4.1 | 31/60; 51.7 | 100.0 | 96.8 | 0.78 (0.57–0.92) | 0.87 (0.37–0.95) |
| DeepSeek V3 | 23/60; 38.3 | 95.0 | 82.6 | 0.88 (0.72–0.98) | 0.98 (0.48–1.00) |
| Gemini 2.0 Flash | 19/60; 31.7 | 85.0 | 94.7 | 0.87 (0.68–0.98) | 0.93 (0.42–0.99) |
| Grok-3 | 20/60; 33.3 | 100.0 | 100.0 | 1.00 (1.00–1.00) | 1.00 (0.50–1.00) |
| Llama 3.3 70B Instruct | 26/60; 43.3 | 95.0 | 88.5 | 0.83 (0.62–0.95) | 0.92 (0.43–0.98) |
| GPT-5 Nano | 19/60; 31.7 | 95.0 | 100.0 | 0.97 (0.91–1.00) | 0.98 (0.48–1.00) |

**Legend: Decision pattern:** SCORE2 withheld = proportion of vignettes where the model flagged SCORE2 as not applicable (n/N; %). Reason provided = proportion of those decisions accompanied by a stated rationale. **Reasoning quality:** Evaluated among reason-provided cases only. Valid Reason = rationale citing a prespecified guideline contraindication (atherosclerotic cardiovascular disease, diabetes mellitus, chronic kidney disease, or familial hypercholesterolaemia). Invalid reasoning — not shown as a separate column but calculable as 100 minus Valid Reason % — comprised citations of other cardiovascular conditions not qualifying as contraindications, or unrelated non-cardiovascular factors. **Condition identification:** Multilabel extraction performance within true exception cases only (vignettes where SCORE2 was correctly not applied). Micro-F1 reflects overall label-level accuracy weighted by condition frequency; Macro-F1 gives equal weight to each of the four condition classes regardless of prevalence. Both metrics reported with 95% bootstrap confidence intervals. Models are ordered by quadratic-weighted κ from the primary three-class ESC risk classification analysis.

## **Supplementary Table S12.** **Numeric agreement with SCORE2**

| **Model** | **N** | **MAE** | **RMSE** | **Bias** | **LoA lower** | **LoA upper** | **CCC** |
| --- | --- | --- | --- | --- | --- | --- | --- |
| **GPT-4o** | 40 | 2.27  (1.53, 3.04) | 3.02  (2.03, 3.79) | -0.89  (-1.89, 0.11) | -6.61  (-8.23, -4.43) | 4.84  (2.41, 6.91) | 0.65  (0.44, 0.79) |
| **GPT-5** | 33 | 1.90  (1.22, 2.74) | 2.54  (1.48, 3.55) | -1.56  (-2.48, -0.80) | -5.54  (-7.58, -3.21) | 2.42  (1.14, 3.28) | 0.71  (0.42, 0.89) |
| **GPT-4.1** | 40 | 2.29  (1.70, 2.93) | 2.78  (2.09, 3.42) | -1.69  (-2.58, -0.76) | -6.07  (-7.30, -4.46) | 2.69  (0.81, 4.49) | 0.66  (0.45, 0.80) |
| **Claude Sonnet 4.5** | 18 | 1.22  (0.53, 2.29) | 1.91  (0.63, 3.09) | -0.97  (-2.12, -0.24) | -4.29  (-6.69, -1.34) | 2.35  (0.41, 2.99) | 0.83  (0.48, 0.97) |
| **Gemini 2.5 Pro** | 27 | 1.29  (0.69, 2.15) | 1.98  (0.98, 3.01) | -1.05  (-1.97, -0.38) | -4.41  (-6.54, -2.18) | 2.31  (1.11, 3.01) | 0.79  (0.51, 0.94) |
| **Claude Opus 4.1** | 29 | 1.67  (0.91, 2.68) | 2.45  (1.17, 3.63) | -1.31  (-2.44, -0.46) | -5.45  (-7.80, -2.58) | 2.83  (1.28, 3.69) | 0.65  (0.28, 0.90) |
| **DeepSeek V3** | 36 | 2.38  (1.52, 3.29) | 3.13  (2.08, 4.02) | -2.20  (-3.12, -1.29) | -6.63  (-8.25, -4.49) | 2.24  (1.08, 3.24) | 0.51  (0.27, 0.73) |
| **Gemini 2.0 Flash** | 38 | 3.16  (2.44, 3.87) | 3.56  (2.86, 4.19) | -3.11  (-3.84, -2.34) | -6.56  (-7.51, -5.36) | 0.34  (-1.06, 1.27) | 0.43  (0.28, 0.54) |
| **Grok-3** | 40 | 1.92  (1.26, 2.63) | 2.55  (1.79, 3.21) | -1.49  (-2.34, -0.65) | -5.60  (-6.85, -3.93) | 2.62  (1.43, 3.62) | 0.59  (0.31, 0.78) |
| **Llama 3.3 70B Instruct** | 31 | 2.68  (1.98, 3.43) | 3.36  (2.39, 4.22) | -1.93  (-3.02, -0.77) | -7.41  (-9.05, -5.20) | 3.55  (1.49, 5.41) | 0.56  (0.32, 0.73) |
| **GPT-5 nano** | 31 | 2.23  (1.64, 2.84) | 2.62  (2.00, 3.16) | -2.07  (-2.73, -1.40) | -5.28  (-6.06, -4.13) | 1.14  (0.06, 1.99) | 0.59  (0.41, 0.71) |

**Legend.** Values are percentage points. MAE = mean absolute error; RMSE = root mean square error; Bias = mean (Model − Gold); LoA = 95% Bland–Altman limits of agreement; CCC = Lin’s concordance correlation coefficient. Rows ordered by κw from the primary classification analysis.

# **Supplementary Appendices (Section III)**

## **Supplementary Appendix S1. Standardized Prompt Template**

This appendix provides the complete Portuguese and English language prompts used for querying all LLMs. Each vignette was submitted using this template, with the text inserted in place of [Vignette]. The format, structure, and variable naming conventions were identical across all models.

| **PROMP RISCO CV PORTUGUÊS**  Função: És um assistente virtual especializado em cardiologia preventiva que apoia a consulta médica.  Objetivo: Analisar a vinheta clínica abaixo, extrair de forma estruturada todos os fatores relevantes para avaliar o risco cardiovascular e calcular o risco segundo as recomendações da Sociedade Europeia de Cardiologia de 2021 (ESC 2021), para a Prevenção Cardiovascular, em países de risco moderado (Portugal).  **[Vinheta]**  **1. Extração estruturada dos fatores de risco:**  - Preenche uma tabela em Markdown com os fatores de risco necessários ao cálculo do risco cardiovascular  - Para cada fator: Valor (tal como aparece na vinheta ou indica “desconhecido”); Unidade se aplicável (mmHg, mg/dL, anos);  - Mantém exatamente o mesmo nome de cada variável indicado na lista-referência abaixo.  - Lista-referência (não alterar nomes):  [**Fator de Risco** -> Valores; Idade -> Numérico; Género->Masculino; Feminino; Tabagismo -> Não fumador; Ex-Fumador; Fumador; Pressão Arterial Sistólica -> Numérico; Colesterol Total -> Numérico, Colesterol HDL -> Numérico, Colesterol não HDL (se necessário calcular) -> Numérico.]  **2. Estratificação de risco segundo recomendações da sociedade europeia de cardiologia 2021 (2021 ESC Guidelines on cardiovascular disease prevention in clinical practice)**  - Verifica se o SCORE2 é aplicável (Sim / Não).  - Se existir algum critério de exceção que impeça a aplicação do SCORE2, indica-o e classifica o doente de acordo com os grupos de risco definidos nas recomendações ESC 2021 (exceções possíveis: Diabetes Mellitus, Dislipidemia Familiar, Doença Renal Crónica, Doença Cardiovascular Aterosclerótica).  - Sempre que aplicável, calcula o SCORE2 (risco cardiovascular a 10 anos, em percentagem), utilizando exclusivamente: 1) os fatores de risco identificados no ponto 1; 2 usando a calculadora online oficial da Sociedade Europeia de Cardiologia para países de risco moderado https://heartscore.escardio.org/Calculate/quickcalculator.aspx?model=moderate;  3) em alternativa, usando a tabela SCORE2 para países de risco moderado, constante nas recomendações; 4) não devem ser utilizadas aproximações nem estimativas baseadas em julgamento clínico subjetivo para determinar o SCORE2.  - De acordo com o resultado do SCORE2, identifica a categoria de risco, tendo em conta a idade (Baixo-a-Moderado; Alto; Muito-Alto)  - Indica o grau de confiança da decisão sobre categoria de risco indicada no ponto anterior (Escolhe apenas uma categoria -> Baixa, Intermédia, Alta, Muito-Alta).  - Apresenta as saídas numa tabela Markdown: \| SCORE2 Aplicável \| Exceção (se não aplicável) \| SCORE2 (%)\| Categoria Risco \| Grau de Confiança  **3. Breve explicação clínica**  Máximo 150 palavras. Realça os fatores que mais contribuíram para a classificação de risco (p. ex. HTA mal controlada, tabagismo, LDL elevado, modificadores de risco, etc.).  **4. Extracção estruturada dos fatores de risco:**  - Preenche uma tabela Markdown com as duas categorias fixas: “Fatores de Risco” e “Modificadores de Risco”.  - Para cada variável apresenta o valor tal como aparece na vinheta ou “desconhecido”; inclui unidade se aplicável (mmHg, mg/dL, anos);  - Mantém exatamente o mesmo nome de cada variável indicado na lista-referência abaixo.  - Quando mais do que uma opção é possível (p. ex. Modificadores de Risco), lista-as separadas por “;”.  - Lista-referência (não alterar nomes):  [**Fator de Risco** -> Valores; Idade -> Numérico; Género->Masculino; Feminino; Tabagismo -> Não fumador; Ex-Fumador; Fumador; Hipertensão Arterial -> Sim, Não; Pressão Arterial Sistólica -> Valor; Pressão Arterial Diastólica -> Valor; Dislipidemia -> Sim, Não; Colesterol Total -> Numérico; Colesterol HDL -> Numérico; Triglicéridos -> Numérico; Colesterol LDL -> Numérico; Colesterol não HDL (se necessário calcular) -> Numérico; Diabetes Mellitus-> Sim, Não  **Modificadores de Risco** -> Valores; Pode selecionar mais do que um -> Score de Cálcio elevado; Score de Cálcio = 0; Placas Carótidas; Obesidade; Pre-Diabetes; História Familiar de doença cardiovascular aterosclerótica; Lp (a) aumentada; Rigidez Arterial aumentada; PCR alta sensibilidade elevada; Doença Inflamatória Crónica; Infecção HIV; Sindrome Apneia Obstrutiva do Sono; Esteatose Hepática Não Alcoólica; Disfunção erétil; Menopausa precoce; Antecedentes de Gravidez com complicações (Pre-eclampsia, Hipertensão arterial ou diabetes); Doença pulmonar obstrutiva crónica (DPOC); Cancro; Outro (especificar)]  **5. Formato geral**  - Usa Markdown; não incluas texto fora das secções acima  - Quando um valor não se encontra nos dados, escreve “desconhecido”  - Usa sempre “,” como separador decimal e “.” para milhares (padrão português)  **6. Criação de um json file**  - No final, cria um json file que sumarize as informações, com a seguinte estrutura:  json_file ={  "Risco':{'SCORE2 Aplicável': '..', 'Exceção': '..','Categoria': '..', 'SCORE2': '..', ‘confiança’: ’..’ },"  "Fatores de risco':{'Fator1':'valor1','Fator2':'valor2', …}"  "Modificadores de risco' : 'FactorM1:'valorM1', 'FactorM2': 'valorM2'}}." |
| --- |
| **PROMPT CV RISK ENGLISH**  **Function**: You are a virtual assistant specialized in preventive cardiology that supports clinical decision-making in medical consultations.  **Objective**: To analyze the clinical vignette below, extract in a structured manner all relevant factors for cardiovascular risk assessment, and calculate risk according to the 2021 European Society of Cardiology Guidelines (ESC 2021) for cardiovascular prevention in moderate-risk countries (Portugal).  **[Vignette]**  **1. Structured extraction of risk factors:**  - Fill in a Markdown table with all risk factors required for cardiovascular risk calculation  - For each factor: provide the value as presented in the vignette or “unknown”; include units where applicable (e.g., mmHg, mg/dL, years).  Use exactly the same variable names as indicated in the reference list below.  - **Reference list** (do not change names):  [Risk Factor -> Values; Age -> Numeric; Gender -> Male; Female; Smoking -> Non-smoker; Former Smoker; Smoker; Systolic Blood Pressure -> Numeric; Total Cholesterol -> Numeric; HDL-Cholesterol -> Numeric; non-HDL Cholesterol (calculate if necessary) -> Numeric].  **2. Risk stratification according to the 2021 ESC Guidelines on cardiovascular disease prevention in clinical practice**  - Check whether SCORE2 is applicable (Yes / No).  - If any exception criteria are present that preclude SCORE2 use, specify them and classify the patient based on ESC 2021 recommended risk groups (possible exceptions: Diabetes Mellitus, Familial Dyslipidemia, Chronic Kidney Disease, Atherosclerotic Cardiovascular Disease).  - When applicable, calculate the SCORE2 (10-year cardiovascular risk, in percentage), using only: 1)The risk factors identified in section 1; 2) the official online calculator for moderate-risk countries provided by the European Society of Cardiology:  https://heartscore.escardio.org/Calculate/quickcalculator.aspx?model=moderate; 3)  Alternatively, use the SCORE2 risk tables for moderate-risk countries as published in the ESC 2021 Guidelines; 4) do not use approximations or clinical judgment-based estimates for SCORE2 calculation.  - Based on the SCORE2 result, determine the risk category, taking age into account (Low-to-Moderate; High; Very High).  - Indicate the confidence level of the assigned risk category (choose one only: Low, Moderate, High, Very High).  - Present the output in a Markdown table as follows: \| SCORE2 Applicable \| Exception (if not applicable) \| SCORE2 (%) \| Risk Category \| Confidence Level  **3. Brief Clinical Explanation**  Provide a brief clinical summary (maximum 150 words) highlighting the main factors that contributed to the assigned risk classification (e.g., uncontrolled hypertension, smoking, elevated LDL, presence of risk modifiers, etc.).  **4. Structured extraction of risk factors:**  - Fill in a Markdown table with two fixed categories: “Risk Factors” and “Risk Modifiers”.  - For each variable, report the value as shown in the vignette or “unknown”; include units, where applicable.  - Maintain exactly the same variable names listed in the reference list below.  - If more than one option applies (e.g., multiple risk modifiers), list them separated by semicolons (;).  **Reference list** (do not alter names):  [Risk Factor -> Values; Age -> Numeric; Gender -> Male, Female; Smoking -> Non-smoker, Former Smoker, Smoker; Arterial Hypertension -> Yes, No; Systolic Blood Pressure -> Numeric; Diastolic Blood Pressure -> Numeric; Dyslipidemia -> Yes; No; Total Cholesterol -> Numeric; HDL-Cholesterol -> Numeric; Triglycerides -> Numeric; LDL-Cholesterol -> Numeric; non-HDL Cholesterol -> Numeric; Diabetes Mellitus -> Yes, No  Risk Modifiers -> Values; More than one can be selected -> Elevated Coronary Calcium Score; Calcium Score = 0; Carotid Plaques; Obesity; Pre-Diabetes; Family History of atherosclerotic cardiovascular disease (ASCVD); Elevated Lp(a); Increased Arterial Stiffness; Elevated High-sensitivity CRP; Chronic Inflammatory Disease; HIV Infection; Obstructive Sleep Apnea (OSA); Non-Alcoholic Fatty Liver Disease; Erectile Dysfunction; Premature Menopause; Past history of Pregnancy Complications (Preeclampsia, Hypertension, or Diabetes); Chronic Obstructive Pulmonary Disease (COPD); Cancer; Other (specify)]  **5. General Formatting**  - Use Markdown; do not include any text outside the sections above.  - If a value is not available, write “unknown”.  - Always use a comma (,) as a decimal separator and a dot (.) for thousands (Portuguese format).  **6. Json File Creation**  - In the end, create a json file that sumarizes the details with the following structure:  json_file ={  "Risk:{' SCORE2 Applicable': '..', ' Exception': '..',‘Category: '..', 'SCORE2': '..', ‘Confidence Level: ’..’ },"  "Risk Factors:{'Factor1':'value1','Factor2':'value2', …}  "Risk Modifiers: {FactorM1: 'ValueM1', FactorM2: 'valueM2', ...}}." |

## **Supplementary Appendix S2. Example simulated outpatient vignette**

Full vignette text in Portuguese and English as used in the study. The full set of vignettes is available at the Open Science Framework project (DOI:10.17605/OSF.IO/J2ZK9).

| **Portuguese Version**  *Homem 48, anos, guarda prisional. Veio encaminhado da consulta do médico de família por hipertensão arterial.*  *Sem dor torácica, síncope, palpitações ou cansaço. Quando interrogado, refere roncopatia e sonolência diurna (embora esposa não note apneias).*  *Sem antecedentes cardiovasculares pessoais ou familiares. Nega tabagismo. Consumo ocasional de bebidas alcoólicas. Não faz qualquer medicação. Refere que o seu posto de trabalho envolve alguma actividade física, mas não faz activamente desporto.*  *Exame objectivo: peso 94Kg (IMC 28.2); pressão arterial 161/102mmHg, frequência cardíaca 81bpm.*  *Analiticamente, apresenta uma creatinina 1.4mg/dL, glicémia em jejum 104mg/dL, HbA1c 6.2%, triglicerideos 197mg/dL, colesterol total 231mg/dL, colesterol HDL 43mg/dL, colesterol LDL 149mg/dL, lipoproteína (a) 29mg/dL, ácido úrico 7.5mg/dL.*  *Realizou um electrocardiograma e um ecocardiograma, sem alterações valorizáveis. MAPA confirmou HTA (média 24h 138-86mmHg) com perfil não-Dipper. Rigidez arterial com VOP 9.3m/s.* |
| --- |
| **English Version**  *48-year-old male, correctional officer. Referred from his primary care physician for hypertension.*  *Denies chest pain, syncope, palpitations, or fatigue. On review of systems, reports snoring and daytime sleepiness (although his wife has not noted apneas).*  *No personal or family history of cardiovascular disease. Denies tobacco use. Occasional alcohol consumption. Takes no medications. Reports that his work involves some physical activity but does not actively practice sports.*  *Physical examination: weight 207 lbs (BMI 28.2); blood pressure 161/102 mmHg; heart rate 81 bpm.*  *Laboratory data: creatinine 1.4 mg/dL; fasting glucose 104 mg/dL; HbA1c 6.2%; triglycerides 197 mg/dL; total cholesterol 231 mg/dL; HDL cholesterol 43 mg/dL; LDL cholesterol 149 mg/dL; lipoprotein(a) 29 mg/dL; uric acid 7.5 mg/dL.*  *He underwent an electrocardiogram and echocardiogram, both without significant findings. Ambulatory blood pressure monitoring confirmed hypertension (24-hour average 138/86 mmHg) with a non-dipper pattern. Arterial stiffness with pulse wave velocity of 9.3 m/s.* |
